# Bidirectional integrin β1 activation synergizes neurovascular coupling and enhances bone regeneration

**DOI:** 10.1038/s41467-026-74071-3

**Published:** 2026-06-12

**Authors:** Fan Wu, Yanxin An, Yuqing Zhao, Jiazhi Yu, Gaoyi Wu, Franklin R. Tay, Yang Jiao, Jing Wang

**Affiliations:** 1 State Key Laboratory of Oral & Maxillofacial Reconstruction and Regeneration, National Clinical Research Center for Oral Diseases, Shaanxi Engineering Research Center for Dental Materials and Advanced Manufacture, Department of Oral Implants, School of Stomatology, The Fourth Military Medical University, Xi’an, P. R. China; 2 Department of General Surgery, The First Affiliated Hospital of Xi’an Medical University, Xi’an, P. R. China; 3 School of Stomatology, Heilongjiang Key Lab of Oral Biomedicine Materials and Clinical Application, Experimental Center for Stomatology Engineering, Jiamusi University, Jiamusi, P. R. China; 4 The Graduate School, Augusta University, Augusta, GA USA; 5 Department of Stomatology, The Seventh Medical Center of PLA General Hospital, Beijing, P. R. China

**Keywords:** Tissue engineering, Biomedical materials, Bioinspired materials

## Abstract

Reconstruction of large segmental bone defects remains challenging because current grafting strategies often fail to coordinate angiogenesis, neurogenesis, and osteogenesis. Here we developed a functional scaffold (peptide/Talin1 plasmid/PLA–HA/GelMA, PTPG) capable of simultaneously delivering peptides and Talin1 plasmids. We hypothesized that this scaffold enables neurovascularized bone regeneration through bidirectional activation of integrin β1 (ITGB1). The REDV–IKVAV (Arg-Glu-Asp-Val-Gly-Gly-Gly-Ile-Lys-Val-Ala-Val) peptide triggers “outside-in” ITGB1 signaling in endothelial and Schwann cells, while Talin1 plasmid-mediated “inside-out” activation. This PTPG scaffold synergistically enhances cell proliferation, migration and secretion, which are eliminated by ITGB1 silencing. In vivo, PTPG scaffold promotes aligned neurovascular networks guiding bone deposition. Single-cell RNA sequencing demonstrates enrichment of endothelial H-type signatures and repair-associated Schwann cell phenotypes, with activation of ITGB1–focal adhesion kinase–paxillin signaling. Collectively, this scaffold integrates structural support with peptide and genetic cues to promote coordinated angiogenesis, neurogenesis, and osteogenesis, offering a promising strategy for functional bone regeneration.

## Abstract (web summary)

Large bone defect repair needs coordinated neurovascular and osteogenic processes. Here, the authors engineer a scaffold to co-delivering peptide and plasmids to bidirectionally activate integrin β1, promoting aligned neurovascular networks that guide bone deposition for bone repair.

## Introduction

Large segmental bone defects caused by trauma, infection, or tumor resection represent a significant clinical challenge, with more than two million patients worldwide at risk of limb amputation each year due to the absence of effective regenerative solutions^1,2^. Conventional approaches, such as autografts and allografts, are limited by donor availability, morbidity, and immune rejection^3,4^. Tissue-engineered scaffolds represent promising alternatives, yet most traditional designs support only osteoconduction or single-factor delivery, failing to recapitulate the neurovascular–osteogenic triad essential for large segmental bone regeneration^5,6^. Emerging evidence suggests that neurogenesis and angiogenesis are not merely concomitant processes but active regulators that critically drive osteogenic initiation and progression^6–8^. Designing scaffolds that orchestrate the interplay between angiogenesis, neurogenesis, and osteogenesis is essential to advancing bone tissue engineering.

Integrins are transmembrane receptors that mediate adhesion between cells and the extracellular matrix (ECM)^9^. These receptors also regulate directional migration and are essential for neurovascular regeneration and bone healing^10,11^. Integrin β1 (ITGB1) on endothelial cells (ECs) binds to ECM proteins including fibronectin and laminin to promote migration, tubulogenesis, and vascular stabilization^12^. In neural cells, integrin-mediated activation of kinases such as focal adhesion kinase (FAK) directs axonal growth and supports neurite extension^13^. In bone tissue, integrins interact with ECM components such as collagen and osteopontin to stimulate osteogenic differentiation, mineralization, and matrix organization^14^. These diverse functions identify integrins as important molecular targets in regenerative scaffold design.

Integrin activation is regulated through bidirectional signaling. “Outside-in” activation is initiated by ligand binding to the extracellular domain, which induces conformational changes and intracellular signaling cascades^15^. Short ECM–derived peptides, including Arg-Glu-Asp-Val (REDV) and Cys-Gly-Arg-Gly-Asp-Asp-Val-Cys-NH2 (LXW7) with vasculotropic activity, or Ile-Lys-Val-Ala-Val (IKVAV) and Tyr-Ile-Gly-Ser-Arg (YIGSR) with neuroactive properties, can mimic native ligands and promote integrin-mediated adhesion and migration^16–19^. Both REDV and LXW7 enhance endothelial recruitment and vascular organization, whereas IKVAV and YIGSR promote neural adhesion, migration, and neurite extension. The incorporation of these peptides into biomaterials has been shown to improve neurovascular regeneration. In preclinical models, peptide-functionalized scaffolds enhanced recruitment of osteoblasts, ECs, and nerve cells to defect sites^20,21^.

Unlike “outside-in” activation, “inside-out” activation is regulated by intracellular adaptor proteins such as Talin and kindlin. These adaptor proteins bind to the cytoplasmic tail of integrins and induce a high-affinity conformation^15^. Talin1 activation promotes integrin clustering, strengthens ligand binding, and amplifies downstream signaling. Experimental models have shown that elevated Talin1 expression activated ITGB1 and facilitated neurite extension^22^. Likewise, Talin1-dependent integrin activation regulates vascular endothelial-cadherin localization and EC angiogenesis function^23^. However, most current integrin-targeting biomaterials rely primarily on unidirectional osteogenic regulation through “outside-in” signaling. A central challenge in biomaterials development is to exploit bidirectional activation of integrins to synergistically stimulate cell migration and enable early-stage neurovascular network reconstruction within scaffolds.

**Fig. 1:**
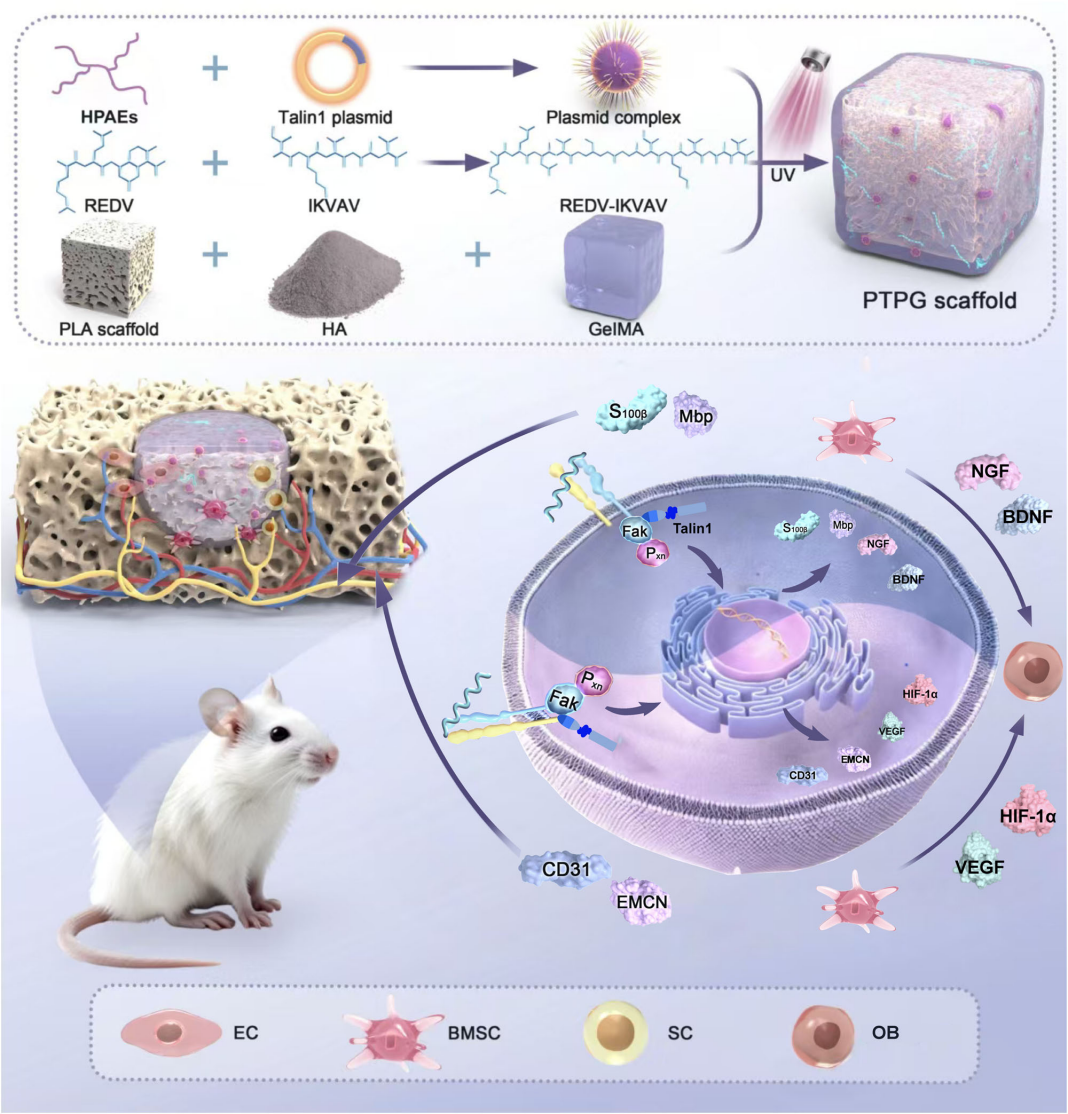
Schematic of an integrin-targeting peptide/Talin1/PLA–HA/GelMA (PTPG) scaffold for neurovascularized bone regeneration. The PTPG scaffold consists of a 3D-printed gradient-porous poly (lactic acid)/hydroxyapatite (PLA–HA) framework combined with a gelatin methacryloyl (GelMA) hydrogel incorporating the chimeric peptide REDV–IKVAV and talin1-encoding plasmids. This design enables bidirectional integrin β1(ITGB1) signaling: peptide ligation mediates “outside-in” activation, while talin1 triggers “inside-out” activation, which modulates integrin affinity in endothelial cell (EC) and Schwann cell (SC), thereby promoting angiogenesis and neurogenesis. Specifically, the activation upregulates the expression of CD31 and EMCN in EC to facilitate vascular formation, and enhances the expression of S100β and MBP in SC to promote nerve regeneration. Additionally, through paracrine signaling, EC secrete HIF-1α and VEGF, while EC release NGF and BDNF, which together support the differentiation of bone marrow-derived mesenchymal stem cell (BMSC) into osteoblasts (OB) and contribute to bone defect repair. HPAEs, hyperbranched poly(β-amino ester)s.

In the present work, we designed a peptide/Talin1 plasmid/PLA–HA/GelMA (PTPG) scaffold to leverage bidirectional ITGB1 activation for neurovascularized bone regeneration (Fig. 1). The construct combines a three-dimensional (3D) printed gradient porous poly (lactic acid)–hydroxyapatite (PLA–HA) framework with a gelatin methacryloyl (GelMA) hydrogel functionalized with a chimeric peptide and Talin1 plasmid delivery. To stimulate “outside-in” signaling, vascular- and neural-specific peptides were conjugated to co-activate ITGB1 in ECs and Schwann cells (SCs), enabling synergistic neurovascular ingrowth. “Inside-out” activation was promoted through hyperbranched poly(β-amino ester)s (HPAEs)-mediated delivery of Talin1-encoding plasmids, enhancing Talin1–integrin binding. We further examined the scaffold’s capacity to provide topographic, biochemical, and mechanical cues that together establish a regenerative microenvironment conducive to functional bone repair. We hypothesized that concurrent activation of ITGB1 through both “outside-in” and “inside-out” signaling pathways synergistically promotes neurovascular network formation, which in turn accelerates osteogenesis and enhances the repair of critical-sized bone defects.

## Results and discussion

### Bidirectional ITGB1 activation enhances neurovascular cell function in vitro

**Fig. 2:**
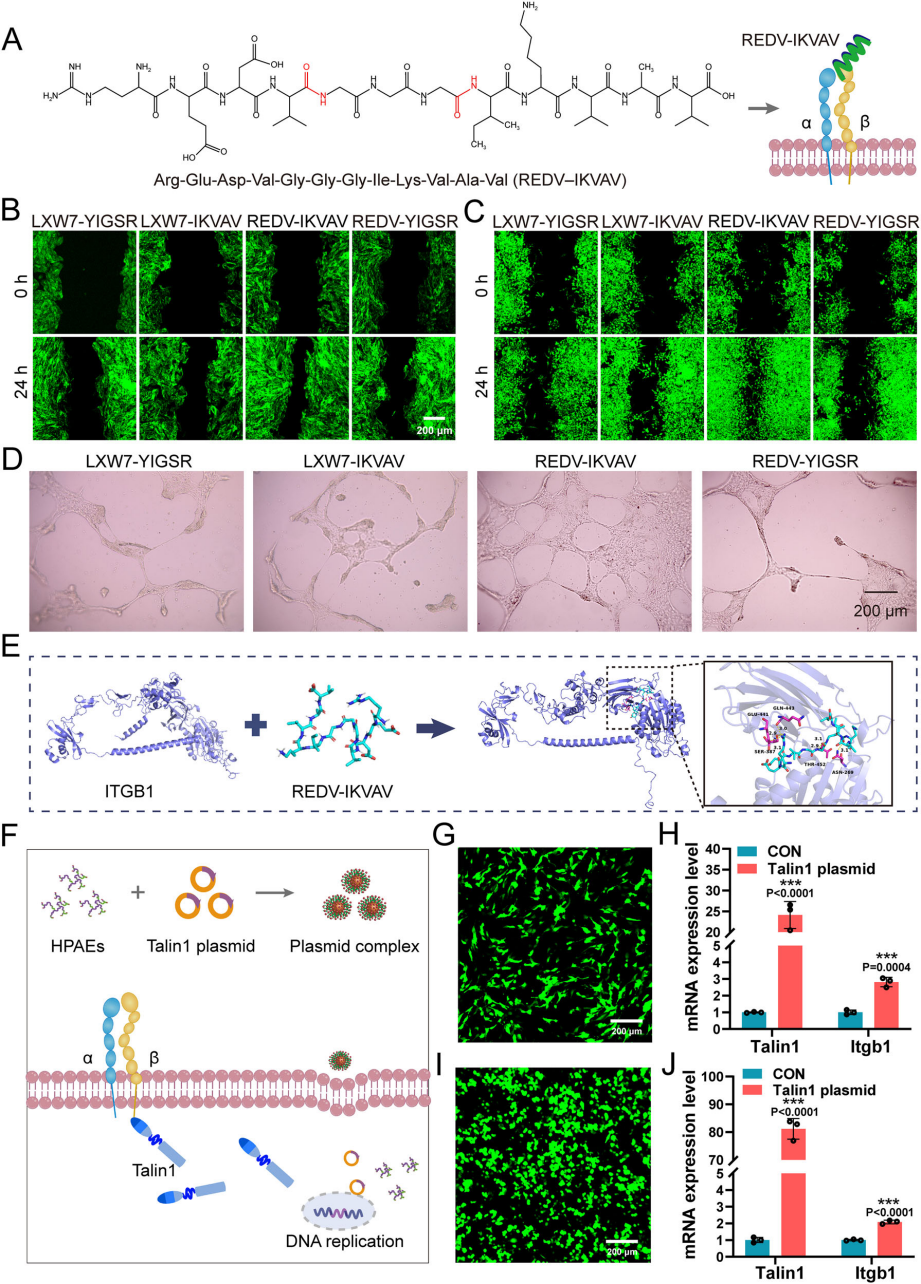
Activation of ITGB1 enhances neurovascular cell function. **A** Chemical structure of Arg-Glu-Asp-Val-Gly-Gly-Gly-Ile-Lys-Val-Ala-Val (REDV–IKVAV). The amide bonds (CONH) formed between carboxyl and amino groups of REDV, GGG and IKVAV; Schematic of the peptide REDV–IKVAV targeting the ITGB1 extracellular domain (“outside-in” activation); **B**, **C** Scratch wound migration assay of human umbilical vein endothelial cells (HUVECs) (**B**) and RSC96 (**C**) cultured with LXW7–YIGSR, LXW7–IKVAV, REDV–IKVAV, and REDV–YIGSR peptides (scale bar = 200 μm). Migration area analysis is shown in Supplementary Fig. 2C; **D** Tube formation assay of HUVECs treated with the indicated peptides (scale bar = 200 μm). Statistical analysis of total length and number of branches is shown in Supplementary Fig. 2D; **E** Molecular docking model of REDV–IKVAV binding to ITGB1 showing hydrogen bonds, ionic interactions, and hydrophobic interactions (binding energy = –5.4 kcal/mol); **F** Schematic of Talin1-mediated “inside-out” integrin activation through binding to the integrin cytoplasmic tail that enhances ligand affinity; **G** Fluorescence images showing the transfection efficiency of Talin1 plasmid in HUVECs; **H** qRT-PCR analysis of Talin1 and Itgb1 expression in the control (CON) and talin1 plasmid transfection groups (***P < 0.001, *n* = 3 independent replicates; scale bar = 200 μm); **I** Fluorescence images showing the transfection efficiency of Talin1 plasmid in RSC96 cells; **J** qRT-PCR analysis of Talin1 and Itgb1 expression in the CON and talin1 plasmid transfection groups (***P < 0.001, *n* = 3 independent replicates; scale bar = 200 μm). Data is represented as the mean ± SD. The *P* value of statistical significance is determined by two-tailed Student’s t-test for two-group comparisons. LXW7–YIGSR (Cyclo-Arg-Gly-Asp-Gly-Gly-Gly-Tyr-Ile-Gly-Ser-Arg), LXW7–IKVAV (Cyclo-Arg-Gly-Asp-Gly-Gly-Gly-Ile-Lys-Val-Ala-Val), REDV–YIGSR (Arg-Glu-Asp-Val-Gly-Gly-Gly-Tyr-Ile-Gly-Ser-Arg). HPAEs, hyperbranched poly(β-amino ester)s.

Vasoactive and neuroactive peptides selectively engage the extracellular domains of integrins, and initiate downstream cascades that mediate “outside-in” signal transduction^22,24^. Covalent coupling of these peptides may synergistically enhance neurovascular repair through integrin-mediated crosstalk, thereby remodeling the neurovascular niche to support functional recovery. To test this hypothesis, we linked vascular-active peptides (REDV^25^, LXW7^16^) and neuroactive peptides (YIGSR^26^, IKVAV^19^) via a triglycine (GGG) linker to preserve peptide conformation and minimize steric hindrance (Fig. 2A). The triglycine linker has been successfully adopted as a tri-amino acid linker^27,28^. Mass spectrometry and reverse-phase high-performance liquid chromatography (RP-HPLC) confirmed the molecular weight and purity of the synthesized chimeric peptides (Supplementary Fig. 1). In the mass spectrometry analysis of REDV–IKVAV, the dominant peak at *m*/*z* 1199.65 corresponds to the singly protonated [M + H]^+^ ion, and the measured molecular weight matches the theoretical value. RP-HPLC further showed that REDV–IKVAV had a purity of 95%, supporting its suitability for subsequent biological experiments.

Biological assays identified REDV–IKVAV as the most effective construct. The CCK-8 assay showed that both REDV–YIGSR and REDV–IKVAV significantly enhanced Human Umbilical Vein Endothelial Cells (HUVEC) and RSC96 proliferation versus the 0 µM group, with no significant differences among the peptide groups (Supplementary Fig. 2A, B). The scratch assay indicated maximal migration in the REDV–IKVAV group (Fig. 2B, C and Supplementary Fig. 2 C). This peptide also significantly promoted tube formation, as indicated by increased total tube length and branch points (Fig. 2D and Supplementary Fig. 2D). In addition, REDV–IKVAV significantly increased ITGB1 protein expression (Supplementary Fig. 2E, F). Based on these findings, REDV–IKVAV was selected for subsequent experiments.

Molecular docking confirmed stable interactions between REDV–IKVAV and ITGB1, with hydrogen bonding, ionic interactions, and hydrophobic forces contributing to a binding energy of –5.4 kcal/mol (Fig. 2E). We further compared the baseline activity of the individual peptides with that of the covalently linked construct. Relative to REDV or IKVAV alone, REDV–IKVAV significantly enhanced HUVEC and RSC96 proliferation and migration and increased ITGB1 expression (Supplementary Fig. 3A–E). These results indicate that covalent linkage confers synergistic bioactivity beyond that achieved with either peptide individually.

In parallel, “inside-out” signaling was evaluated through Talin1 binding to the ITGB1 cytoplasmic tail, which enhances ligand-binding affinity and potentiates cellular functions (Fig. 2F). Previous evidence has demonstrated that Talin1 deficiency leads to widespread hemorrhaging due to impaired integrin activation^29^. Conversely, overexpression of Talin1 has been shown to promote axonal growth^30^. However, due to the large size of the full-length Talin1 plasmid, conventional transfection systems exhibit low efficiency in delivering such oversized plasmids. HPAEs can effectively condense and encapsulate large plasmid constructs, forming stable nanocomplexes that resist lysosomal degradation and enhance delivery efficiency^31^. Fluorescence microscopy confirmed efficient delivery of Talin1 plasmids into ECs and SCs, with associated upregulation of *Talin1* and *Itgb1* expression (Fig. 2G–J). Subsequent functional assays validated the cooperative effect of REDV–IKVAV peptide and Talin1 plasmids in enhancing neurovascular cell functions.

### Fabrication and characterization of PTPG scaffold

**Fig. 3:**
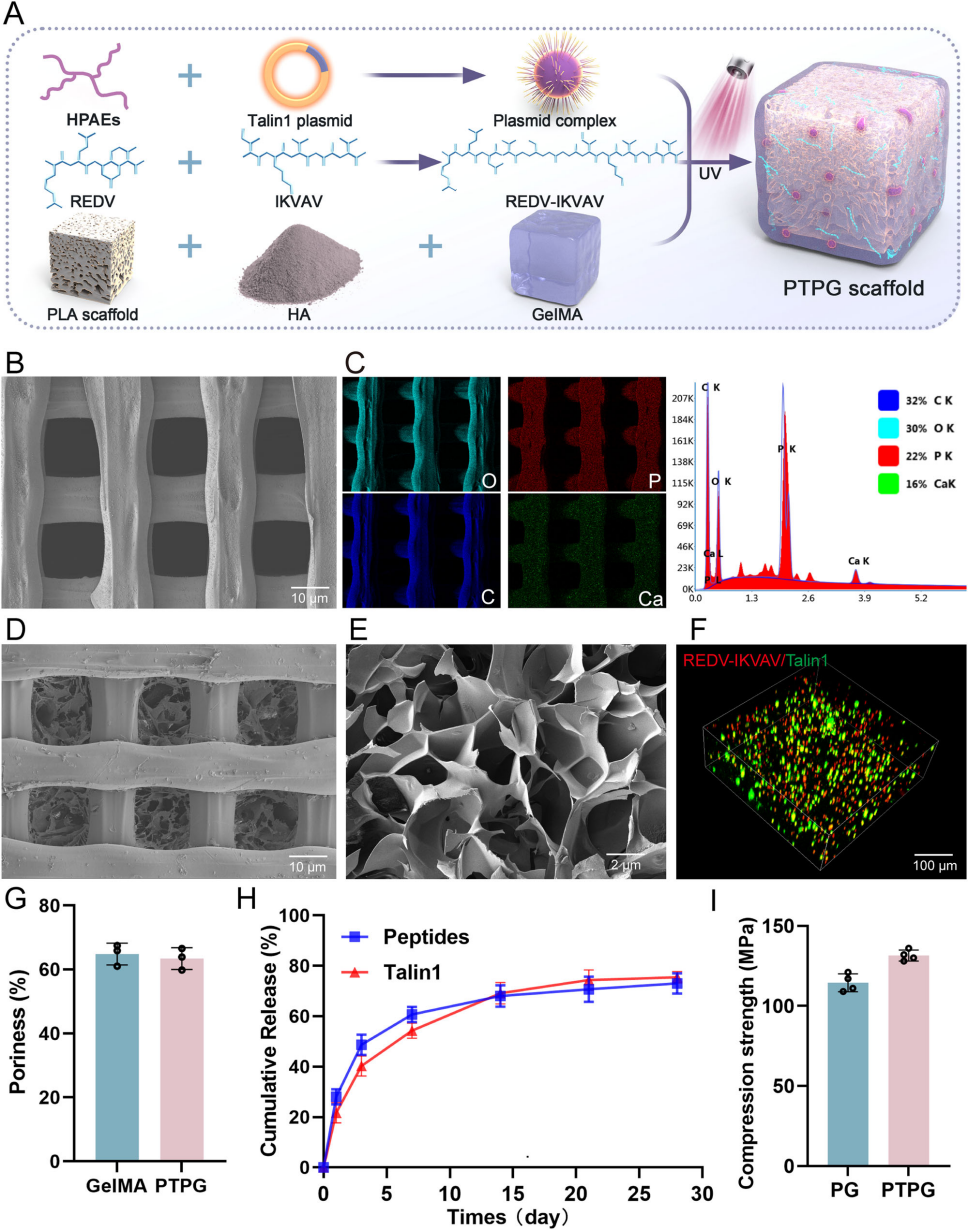
Fabrication and characterization of PTPG scaffold. **A** Schematic illustrating preparation of the PTPG scaffold; **B** SEM image of the PLA–HA scaffold surface (scale bar = 10 μm); **C** Energy-dispersive X-ray elemental mapping (left) and profile (right) of the PLA–HA scaffold showing distribution of C, O, P, and Ca; **D** SEM images of the outer PTPG scaffold (scale bar = 10 μm); **E** SEM image of freeze-dried GelMA hydrogel (scale bar = 2 μm); **F** Laser confocal scanning microscopy showing distribution of REDV–IKVAV and Talin1 plasmid complex in hydrogels (scaler bar = 100 μm); **G** Porosity quantification of GelMA hydrogel and PTPG scaffold (*n* = 3 independent replicates, mean values ± SD); **H** Cumulative in vitro release of REDV–IKVAV peptide and Talin1 plasmid from the PTPG scaffold over 28 days (*n* = 3 independent replicates, mean values ± SD); **I** Compressive strength of PG and PTPG scaffolds (*n* = 4 independent replicates, mean values ± SD). HPAEs, hyperbranched poly(β-amino ester)s; PLA, polylactic acid; HA, hydroxyapatite; PTPG, Peptide/Talin1 plasmid/PLA-HA/GelMA.

Bone repair biomaterials have evolved from physical biomimicry to physiological biomimicry^32^. Native bone exhibits a hierarchical structure consisting of a dense outer layer of cortical bone and a porous inner region of cancellous bone^33^. Because of its biocompatibility and mechanical strength, we fabricated gradient-porous PLA scaffolds by fused deposition modeling 3D printing (Fig. 3A). Hydroxyapatite (HA), the principal inorganic component of bone, was incorporated by dip-coating to improve osteoinductivity^34^. Field-emission scanning electron microscopy (FE-SEM) demonstrated uniform distribution of HA nanoparticles (Fig. 3B and Supplementary Fig. 4A, B). Energy-dispersive X-ray spectroscopy (EDS) mapping confirmed homogeneous elemental signals for C, O, P, and Ca (Fig. 3C). These results were indicative of successful HA integration. Therefore, we constructed a gradient porous scaffold with physical biomimicry to replicate the hierarchical architecture of bone. Some studies also use gradient porous scaffold to repair bone defects. For example, Zhang *et al*. reported that the gradient porous design promotes bone regeneration by providing a stable mechanical environment and facilitating cell ingrowth^35^. Wu et al. demonstrated that mimicking cancellous and compact bone structures results in improved bone regeneration^36^.

In previous studies on physiological biomimicry, the common approach has been to incorporate bioactive components to promote bone regeneration^37^. In our study, by activating ITGB1 on ECs and SCs, we demonstrated the synergistic promotion of neurovascular regeneration, which creates a favorable microenvironment for bone regeneration. Therefore, we functionalized the PLA–HA scaffolds with a GelMA hydrogel co-loaded with REDV–IKVAV chimeric peptide and Talin1 plasmids delivered by HPAEs. We termed this composite scaffold PTPG. Field Emission Scanning Electron Microscope (FE-SEM) revealed a biphasic architecture, with both outer and inner layers displaying a polymeric framework infiltrated by hydrogel (Fig. 3D, E and Supplementary Fig. 4C). After ultraviolet irradiation, all precursor solutions formed stable hydrogels, indicating successful encapsulation of both the peptide and the Talin1 plasmid. Laser scanning confocal microscopy (LSCM) images revealed uniform distribution of the peptide and plasmid throughout the hydrogel matrix (Fig. 3F). Porosity analysis indicated high interconnectivity conducive to cell infiltration, with porosity values of 64.8% for the GelMA hydrogel and 63.4% for the composite scaffold (Fig. 3G).

An in vitro release assay showed that the PTPG scaffold achieved approximately 75% cumulative release and 80% loading efficiency of REDV–IKVAV peptide and Talin1 plasmid (Fig. 3H and Supplementary Fig. 4D). Such an observation is indicative of sustained bioactive signaling. Compression testing revealed a compressive strength of 130 MPa (Fig. 3I), which is sufficient for maintaining stability under physiological loading conditions^38^. We further characterized the structural integrity of the peptide and Talin1 plasmid released from the hydrogel. Circular dichroism spectroscopy confirmed that the secondary structure of the released peptide was consistent with that of the original REDV–IKVAV peptide (Supplementary Fig. 4E). Agarose gel electrophoresis of the released Talin1 plasmid showed a single band, indicating preserved plasmid integrity (Supplementary Fig. 4F). The combination of biomimetic porosity, mechanical robustness, and sustained factor release renders PTPG a multifunctional scaffold capable of supporting neurovascular ingrowth and bone regeneration.

### PTPG scaffold promotes angiogenesis and neurotrophic functions in vitro

**Fig. 4:**
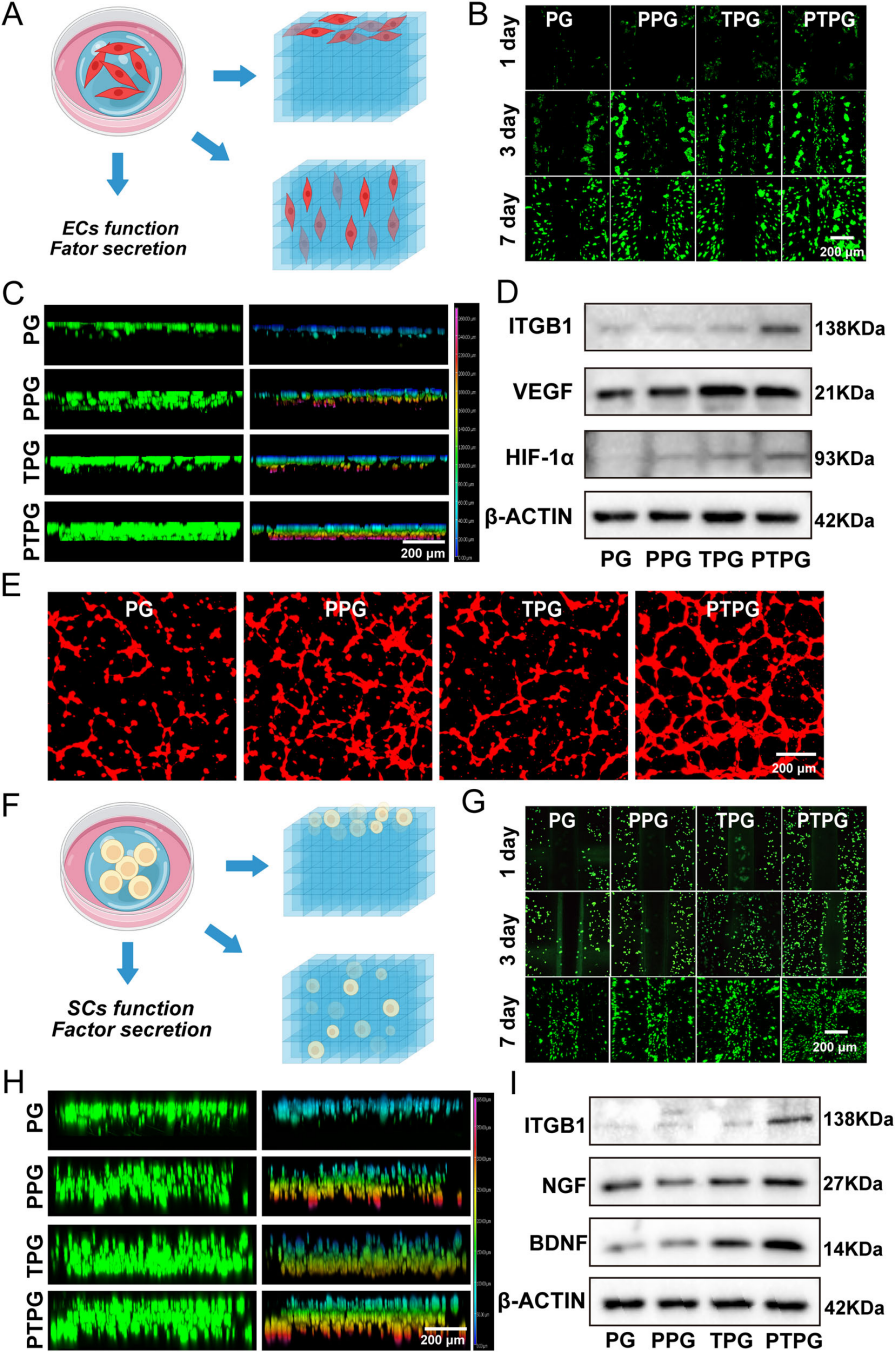
PTPG scaffold enhances neurovascular cell function. **A** Schematic of experimental design for endothelial cell (EC) functional assessment: proliferation, migration and cellular functions; **B** Fluorescence images of HUVECs cultured on different scaffolds for 1, 3, and 7 days (scale bar = 200 μm); **C** Fluorescence and confocal laser scanning microscopy 3D reconstruction showing longitudinal human umbilical vein endothelial cells (HUVECs) migration after 7 days of culture (scale bar = 200 μm); **D** Western blot of ITGB1, VEGF, and HIF-1α expression in HUVECs (*n* = 3 independent replicates). Quantitative analyses are shown in Supplementary Fig. 5D; **E** Tube formation assay of HUVECs cultured with the extracts of four scaffolds (*n* = 3 independent replicates; scale bar = 200 μm). Statistical analysis of total length and number of nodes of tube formation is shown in Supplementary Fig. 5E; **F** Schematic of experimental design for Schwann cell (SC) functional assessment: proliferation, migration and cellular functions; **G** Fluorescence images of RSC96 cells cultured on different scaffolds for 1, 3, and 7 days (scale bar = 200 μm); **H** Fluorescence and confocal 3D reconstruction showing longitudinal RSC96 migration after 7 days of culture (scale bar = 200 μm); **I** Western blot of ITGB1, NGF, and BDNF expression in RSC96 cells (*n* = 3 independent replicates). Quantitative analyses are shown in Supplementary Fig. 5G. The experiment was divided into four groups: PG, PLA-HA/GelMA; PPG, Peptide/PLA-HA/GelMA; TPG, Talin1 plasmid/PLA-HA/GelMA; PTPG, Peptide/Talin1 plasmid/PLA-HA/GelMA.

Angiogenesis is essential for bone regeneration because it facilitates nutrient and oxygen delivery and recruits osteoprogenitor cells through paracrine signaling^39,40^. We therefore evaluated the influence of the scaffolds on ECs (Fig. 4A). CCK-8 assay and fluorescence staining demonstrated sustained EC proliferation in all groups, with significantly accelerated growth in the PTPG group at days 3 and 7 (Fig. 4B and Supplementary Fig. 5A). These findings indicate the biocompatibility and favorable cell-supportive properties of PTPG, which are attributed to the biomimetic microenvironment and controlled release of bioactive cues. 3D reconstruction of LSCM images confirmed deeper ECs migration within PTPG scaffold compared with the PG, PPG, and TPG groups (Fig. 4C). Upregulation of angiogenic markers HIF-1α and VEGF, along with ITGB1, further confirmed that REDV–IKVAV/Talin1-mediated integrin activation amplified angiogenic signaling (Fig. 4D and Supplementary Fig. 5C). This result is consistent with the graded porosity and integrin-specific design of the scaffold. In Matrigel assays, PTPG extracts significantly enhanced tubulogenesis, as indicated by increased tube length, node number, and mesh density (Fig. 4E).

Schwann cells support nerve regeneration and bone repair by secreting neurotrophic factors such as nerve growth factor (NGF) and brain-derived neurotrophic factor (BDNF)^41,42^. Hence, we examined the effect of the scaffolds on RSC96 cells (Fig. 4F). Both CCK-8 assay and fluorescence staining showed the highest SC proliferation in the PTPG group (Fig. 4G and Supplementary Fig. 5B). Confocal reconstruction demonstrated greater vertical migration within PTPG scaffolds than in other groups (Fig. 4H). This finding highlights the synergistic interaction between ITGB1 activation and structural cues. The PTPG scaffold also elicited the strongest upregulation of NGF and BDNF expression (Fig. 4I and Supplementary Fig. 5F), confirming enhanced neurotrophic signaling via ITGB1. Previous studies reported that NGF regulates nerve ingrowth density during bone healing through activation of tropomyosin receptor kinase A^43^, while BDNF enhances bone marrow-derived mesenchymal stem cells (BMSCs) proliferation and migration in response to neuronal signals^44^. Collectively, these results identify PTPG as a bifunctional scaffold that simultaneously stimulates angiogenesis and neurogenesis to create a supportive microenvironment for bone regeneration.

### ITGB1 is essential for PTPG scaffold-mediated neurovascular regeneration

**Fig. 5:**
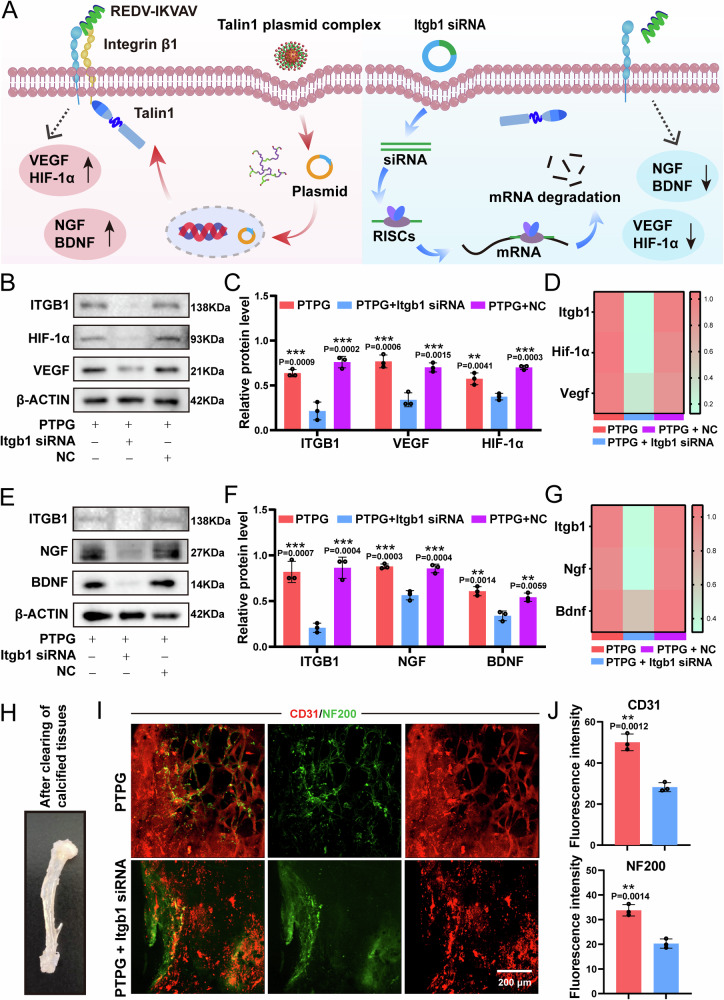
ITGB1 is required for PTPG scaffold-enhanced neurovascular cell function. **A** Schematic illustrating the role of ITGB1 in PTPG scaffold-driven neurovascular regeneration. Under normal conditions, PTPG scaffold activates ITGB1 to promote endothelial cell (EC) angiogenesis and Schwann cell (SC) neurotrophy. ITGB1 silencing disrupts this signaling and impairs neurovascular formation; **B**, **C** Western blot (**B**) and quantitative analyses (**C**) of ITGB1, VEGF, and HIF-1α expression in HUVECs with or without ITGB1 silencing (**P < 0.01, ***P < 0.001, *n* = 3 independent replicates), PTPG, Peptide/Talin1 plasmid/PLA-HA/GelMA; NC, Negative Control; **D** qRT-PCR analysis of Hif-1α, Vegf, and Itgb1 expression in HUVECs with or without ITGB1 silencing (*n* = 3 independent replicates); **E**, **F** Western blot (**E**) and quantitative analyses (**F**) of NGF, BDNF, and ITGB1 expression in RSC96 cells with or without ITGB1 silencing (**P < 0.01, ***P < 0.001, *n* = 3 independent replicates); **G** qRT-PCR analysis of Ngf, Bdnf, and Itgb1 expression in RSC96 cells with or without ITGB1 silencing (*n* = 3 independent replicates); **H** Bone tissue after clearing of calcified tissue; **I** Representative immunofluorescence staining of CD31 and NF200; **J** quantitative analysis of CD31 and NF200 in the defect area (**P < 0.01, *n* = 3 independent replicates; scale bar = 200 μm). Data are presented as mean values ± SD. The *P* value of statistical significance is determined by two-tailed Student’s t-test for two-group comparisons and two-tailed one-way ANOVA with Tukey’s post hoc test for four-group comparisons.

We performed loss-of-function studies using siRNA-mediated knockdown of *ITGB1* to clarify the role of ITGB1 in PTPG-mediated pro-angiogenic and pro-neurotrophic activity (Fig. 5A). qRT-PCR and western blot assays respectively confirmed significant downregulation of *ITGB1* mRNA and protein in siRNA-transfected cells (Fig. 5B–G). In ECs, *ITGB1* silencing impaired angiogenic activity, with markedly reduced VEGF and HIF-1α expression at both transcript and protein levels (Fig. 5B–D). These observations are consistent with reports that ITGB1 blockade suppresses endothelial sprouting^45^. In SCs, *ITGB1* knockdown substantially decreased the secretion and gene expression of NGF and BDNF (Fig. 5E–G). These findings are indicative of the role of ITGB1 in maintaining neurotrophic function on the PTPG scaffold. Although the PLA-GelMA scaffold provides the basic microenvironment for supporting adhesion and proliferation, the induction of angiogenic and neurotrophic responses relies on ITGB1–dependent cell–matrix interactions.

We validated these in vitro findings by in vivo observations using ultrafast immunolabeling and clearing of calcified tissues to visualize neurovascular regeneration (Fig. 5H). The PTPG scaffold-implanted defects exhibited robust angiogenesis and nerve fiber regeneration, with CD31^+^ blood vessels and NF200^+^ nerve fibers showing distinct spatial coordination. This organized neurovascular patterning was absent when *ITGB1* was inhibited (Fig. 5I, J). These results demonstrate that PTPG scaffold-induced enhancement of EC and SC functions depends on ITGB1 specificity and downstream activation. Our result indicated that ITGB1 is a critical mediator, which is supported by previous studies^45^. Overall, these findings establish ITGB1 as a critical molecular target through which PTPG scaffold mediates neurovascularized tissue regeneration.

### PTPG scaffold drives osteogenesis via synergistic neurovascular paracrine signaling in vitro

**Fig. 6:**
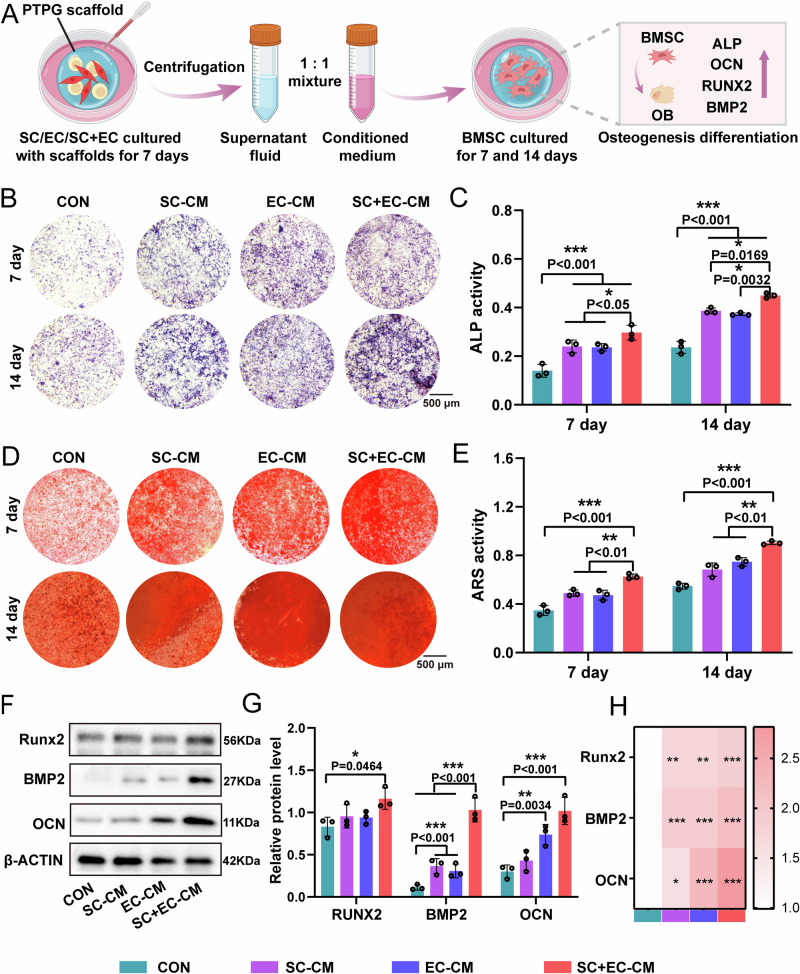
PTPG scaffold enhances osteogenesis through neurovascular paracrine signaling. **A** Schematic of the indirect coculture system used to evaluate the effects of endothelial cell (EC) and Schwann cell (SC) conditioned media (CM) on bone marrow-derived mesenchymal stem cells (BMSCs) differentiate into osteoblast (OB); **B**, **C** Alkaline phosphatase (ALP) staining (**B**) and quantitative activity analysis (**C**) of BMSCs cultured with different CMs for 7 and 14 days (*P < 0.05, ***P < 0.001, *n* = 3 independent replicates, scale bar = 500 μm); **D**, **E** Alizarin red S staining (**D**) and quantification of mineralized nodules (**E**) in BMSCs cultured with different CMs for 7 and 14 days (**P < 0.01, ***P < 0.001, *n* = 3 independent replicates, scale bar = 500 μm); **F**, **G** Western blot (**F**) and quantitative analyses (**G**) of RUNX2, BMP2, and OCN expression in BMSCs cultured with different CMs on day 7 (*P < 0.05, **P < 0.01, ***P < 0.001, *n* = 3 independent replicates); **H** qRT-PCR analysis of Runx2, Bmp2, and Ocn expression in BMSCs cultured with different CMs (*n* = 3 independent replicates). Data are presented as mean values ± SD. The *P* value of statistical significance is determined by two-tailed one-way ANOVA with Tukey’s post-hoc test.

To directly evaluate this paracrine influence, conditioned medium (CM) was collected from ECs (E-CM), SCs (S-CM), and EC–SC co-cultures (ES-CM), and examined their effects on BMSCs (Fig. 6A). All CM groups significantly enhanced BMSC proliferation relative to the control group, with the strongest mitogenic effect observed for the ES-CM group, suggesting synergistic neurovascular paracrine activity. We then evaluated osteogenic differentiation and mineralization. Alkaline phosphatase and Alizarin red S staining showed minimal osteogenesis in the control group, whereas the E-CM promoted both early ALP activity and late-stage mineral deposition (Fig. 6B–E). S-CM also stimulated osteogenesis, albeit less than E-CM. ES-CM produced the most pronounced effect, with ALP activity exceeding that of E-CM and matrix mineralization reaching maximal levels.

At the molecular level, both E-CM and S-CM increased expression of the osteogenic markers OCN and BMP2, with E-CM inducing higher levels than S-CM (Fig. 6F–H). ES-CM again yielded the highest upregulation, confirming that combined EC and SC paracrine signals synergistically enhance osteogenesis. Liu et al. demonstrated that ECs enhance the osteogenic differentiation of BMSCs by secreting exosomes, constituting a positive feedback loop that promotes osteogenesis–angiogenesis coupling^46^. Lao et al. found that remodeling SCs to enhance their secretory function within the diabetic microenvironment helps maintain BMSC function^47^. These studies confirmed that both ECs and SCs facilitate osteogenic differentiation via paracrine signaling pathways, consistent with our findings. Our results further demonstrate that co-activation of paracrine pathways in ECs and SCs produces a stronger pro-osteogenic effect than their individual activation. However, the underlying mechanisms require further elucidation through single-cell RNA sequencing.

### PTPG scaffold promotes structural and functional bone regeneration in a rat bone defect model

**Fig. 7:**
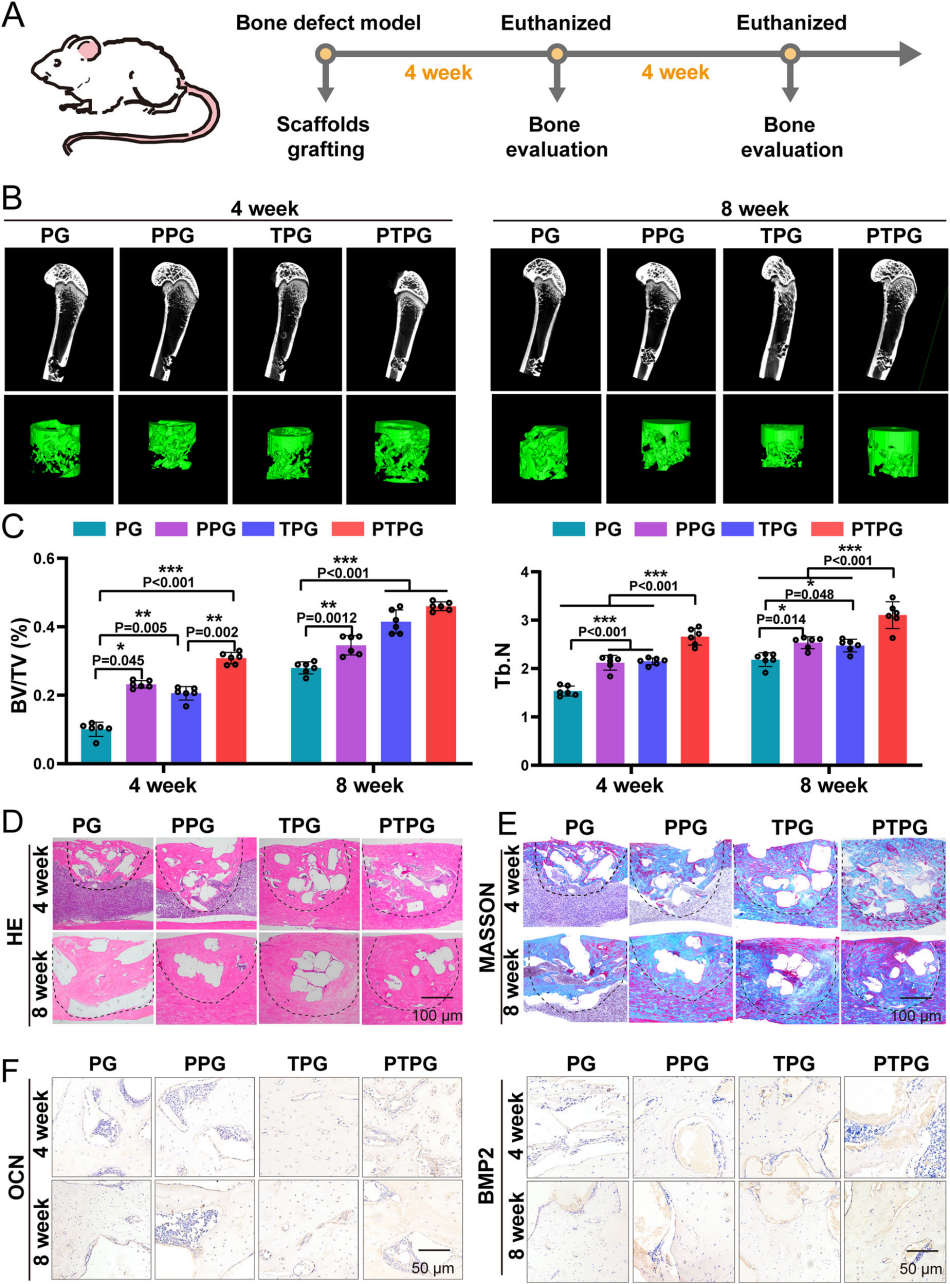
PTPG scaffold promotes bone regeneration in a rat critical-sized femoral defect model. **A** Schematic of experimental design for evaluating bone regeneration in the femoral defect model; **B** Representative 2D axial micro‑CT images and 3D reconstructions of bone defect sites across four experimental groups at 4 and 8 weeks post‑implantation; **C** Quantitative evaluation of bone microarchitecture parameters, including bone volume/total volume (BV/TV) and trabecular number (Tb.N) (*P < 0.05, **P < 0.01, ***P < 0.001, *n* = 6 independent replicates); **D**, **E** Histological sections stained with hematoxylin and eosin (**D**) and Masson’s trichrome (**E**) showing new bone formation at 4 and 8 weeks after implantation (scale bar = 100 μm); **F** Immunohistochemical staining of OCN and BMP2 in the defect area (scale bar = 50 μm). Quantitative analysis of the positive area of BMP2 and OCN are shown in Supplementary Fig. 6. Data are presented as mean values ± SD. The *P* value of statistical significance is determined by two-tailed one-way ANOVA with Tukey’s post-hoc test. PG, PLA-HA/GelMA; PPG, Peptide/PLA-HA/GelMA; TPG, Talin1 plasmid/PLA-HA/GelMA; PTPG, Peptide/Talin1 plasmid/PLA-HA/GelMA.

We evaluated the bone regenerative capacity of the PTPG scaffold in a rat critical-sized femoral defect model (Fig. 7A). Micro-computed tomography revealed distinct differences in new bone formation among the scaffold groups at 4 and 8 weeks post-implantation (Fig. 7B). At 4 weeks, the PG group showed minimal bone regeneration with well-defined defect margins, whereas the PPG and TPG groups supported the formation of limited cancellous and cortical bone. In contrast, the PTPG group exhibited substantial bone deposition, with abundant new tissue and restoration of cortical continuity. By 8 weeks, bone regeneration was further enhanced in all groups, with the PTPG group showing near-complete defect bridging and a well-organized cortical–cancellous structure resembling native bone (Fig. 7C).

Histological evaluation provided further evidence of osteogenic maturation and microstructural restoration. Hematoxylin and eosin and Masson’s trichrome staining demonstrated extensive mature trabecular bone formation in the PTPG group, indicating advanced healing and successful bridging (Fig. 7D, E). Immunohistochemical staining further revealed significantly elevated expression of OCN (a late-stage osteogenic marker) and BMP-2 (a key osteogenic regulator) in the PTPG group (Fig. 7F and Supplementary Fig. 6). These findings demonstrate that the PTPG scaffold exhibits superior osteogenic capacity compared to physical biomimetic scaffolds, due to its synergistic promotion of vascularization, innervation, and bone formation. Nevertheless, little research has been conducted on bone biomimetic scaffolds that incorporate the coordinated functions of the neuro-vascular-bone triad.

### PTPG scaffold promotes coupled neurovascular regeneration in a rat bone defect model

**Fig. 8:**
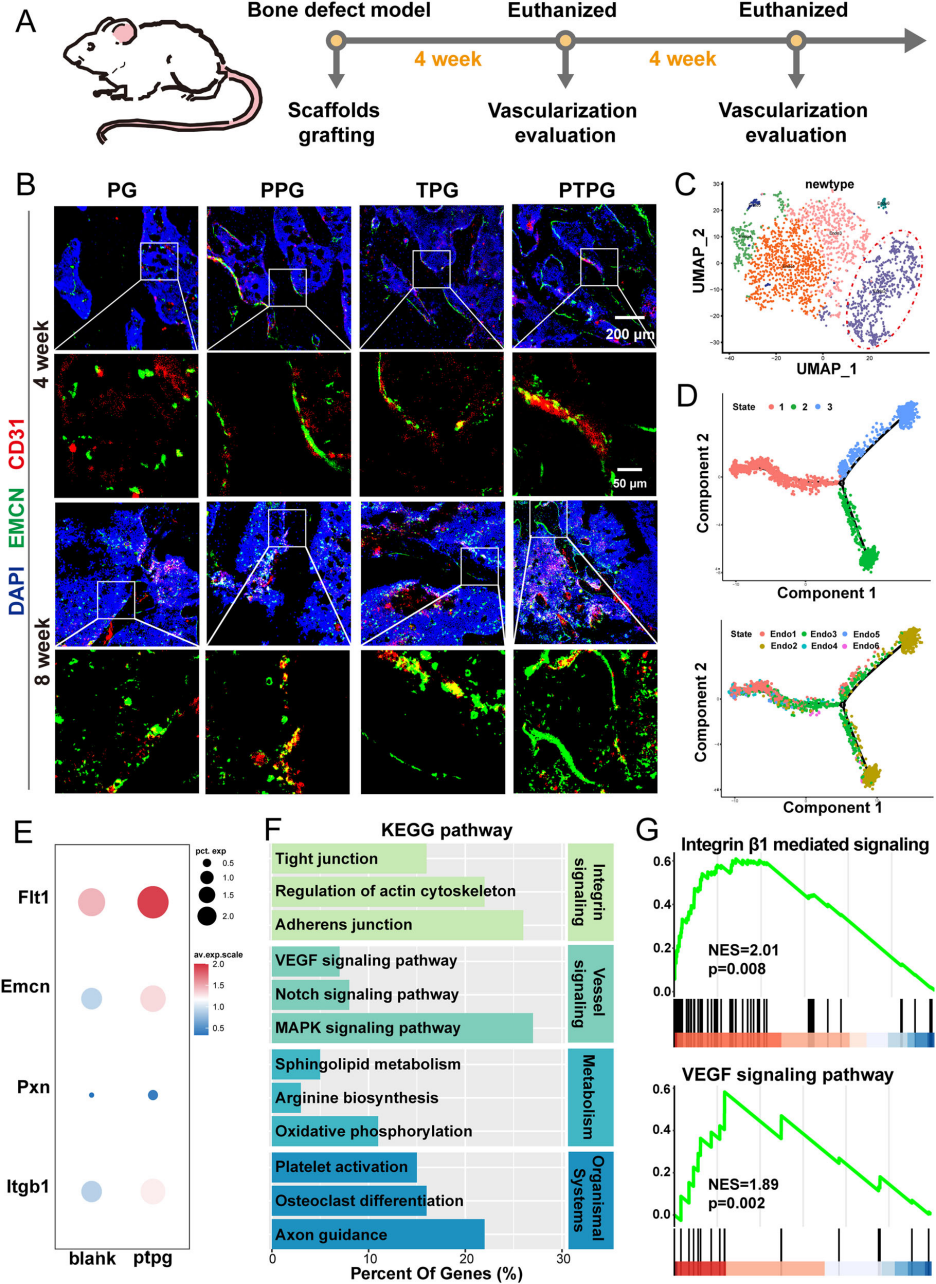
PTPG scaffold promotes functional angiogenesis and EC specification in vivo. **A** Schematic of experimental design for evaluating angiogenesis in the bone defect model; **B** Representative immunofluorescence images of CD31^+^/EMCN^+^ vessels in the defect area at 4 and 8 weeks post-implantation (scale bar = 200 μm). Quantitative analysis of the mean fluorescence intensity of CD31 and EMCN is presented in Supplementary Fig. 7. **C** Uniform Manifold Approximation and Projection (UMAP) visualization of endothelial cell (EC) subclusters derived from single-cell RNA sequencing (scRNA-seq) data; **D** Pseudotime trajectory analysis illustrating EC differentiation dynamics; **E** Dot plot showing the relative expression profiles of angiogenesis- and integrin signaling-related genes (Flt1, Emcn, Pxn, Itgb1) in the bone defect repair microenvironment of the blank and PTPG groups; **F** Kyoto Encyclopedia of Genes and Genomes (KEGG) pathway enrichment analysis of differentially expressed genes in EC. The figure illustrates the core biological pathways enriched by the PTPG scaffold during bone defect repair, including integrin signaling, vessel signaling, metabolism, and organismal systems; **G** Gene Set Enrichment Analysis (GSEA) plot showing significant enrichment of ITGB1-mediated signaling pathway and VEGF signaling pathway, statistical analysis was performed using a permutation test with two-sided testing. NES, Normalized enrichment score; PG, PLA-HA/GelMA; PPG, Peptide/PLA-HA/GelMA; TPG, Talin1 plasmid/PLA-HA/GelMA; PTPG, Peptide/Talin1 plasmid/PLA-HA/GelMA.

**Fig. 9:**
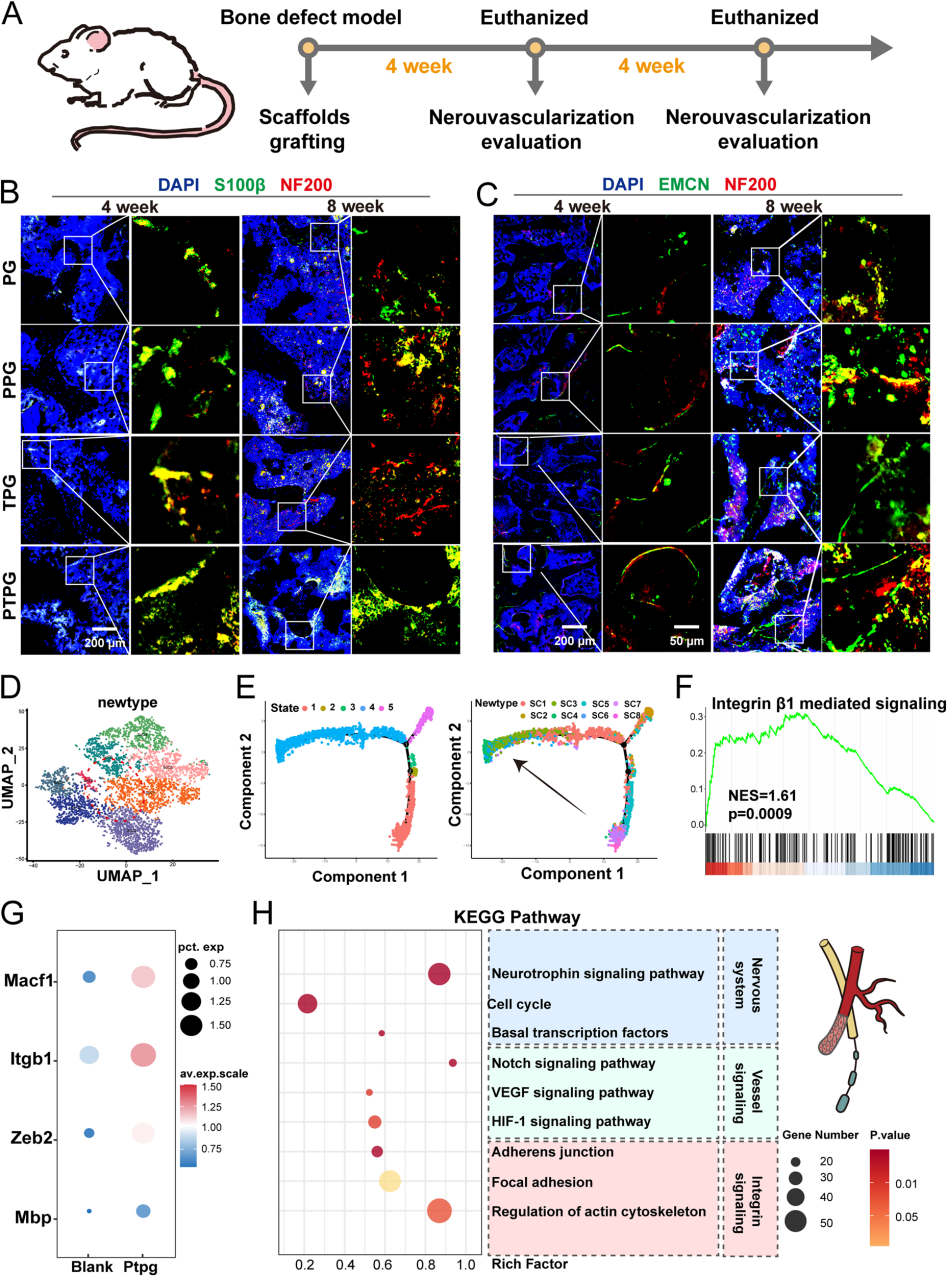
PTPG scaffold promotes coupled neurovascular regeneration and Schwann cell remodeling in vivo. **A** Schematic of experimental setup for evaluating neurovascular regeneration in the bone defect model; **B** Immunofluorescence images of S100β and NF200 showing neural regeneration and myelination within the defect area (scale bar = 200 μm). Quantitative analysis of the mean fluorescence intensity of S100β and NF200 are shown in Supplementary Fig. 8; **C** Co-staining of EMCN and NF200 demonstrating spatial coupling between vasculature and nerve fibers (scale bar = 200 μm); **D** Uniform Manifold Approximation and Projection (UMAP) visualization of Schwann cell (SC) subclusters from single-cell RNA sequencing (scRNA-seq) data; **E** Pseudotime trajectory analysis revealing SC differentiation states; **F** Gene Set Enrichment Analysis (GSEA) enrichment plot for ITGB1-mediated signaling pathway, statistical analysis was performed using a permutation test with two-sided testing; **G** Dot plot showing the relative expression profiles of neurogenesis- and integrin signaling-related genes (Macf1, Itgb1, Zeb2, Mbp) in the bone defect repair microenvironment of the blank and PTPG groups; **H** Top enriched KEGG pathways in SC, including nervous system, vessel signaling, and integrin signaling, statistical analysis was performed using a permutation test with two-sided testing. The right schematic shows blood vessels and nerves in the repair microenvironment. PG, PLA-HA/GelMA; PPG, Peptide/PLA-HA/GelMA; TPG, Talin1 plasmid/PLA-HA/GelMA; PTPG, Peptide/Talin1 plasmid/PLA-HA/GelMA.

We further investigated the ability of the PTPG scaffold to promote vascular and neural ingrowth in the rat femoral defect model (Fig. 8A). Immunofluorescence staining at 4 weeks revealed a significant increase in CD31^+^/EMCN^+^ H-type blood vessels in the PTPG group compared with controls (Fig. 8B and Supplementary Fig. 7). These specialized capillaries are associated with angiogenic–osteogenic coupling^12^. The CD31^+^/EMCN^+^ capillaries were structurally immature but extensively distributed, indicating active initiation of vascularization that supports subsequent osteogenesis^48,49^. By 8 weeks, the PTPG group exhibited a more mature vascular network that was characterized by larger vessel diameter, greater branching, and enhanced CD31^+^/EMCN^+^ co-expression. The result is consistent with a stabilized vasculature capable of sustaining the metabolic demands of regenerated bone^50,51^. Neural regeneration was evaluated using NF200^+^/S100β^+^ dual staining. Robust nerve fiber ingrowth and early myelination were observed within the PTPG group at 4 weeks (Fig. 9B and Supplementary Fig. 8). By 8 weeks, nerve density and myelin maturity were further increased, and integration with newly formed bone was evident. Compared with the other groups, the PTPG group showed significantly higher fluorescence intensity of calcitonin gene-related peptide (CGRP)-positive and tyrosine hydroxylase (TH)-positive signals (Supplementary Fig. 9 and 10). These findings indicate that PTPG promotes regeneration of functional neural subtypes rather than merely general neural structures. The regenerated nerve fibers orchestrate bone regeneration by releasing neurotrophic factors (e.g., NGF, BDNF) and neuropeptides (e.g., SP, CGRP), which directly stimulate osteogenesis and restore skeletal metabolic balance^52,53^. Spatiotemporal analysis of neurovascular coupling with EMCN^+^/NF200^+^ co-staining demonstrated aligned patterns of vessel and nerve distribution in the PTPG group, with nerve fibers frequently extending along nascent vasculature (Fig. 9C). Partial coupling was noted in the PPG group. The combination of REDV–IKVAV peptide and Talin1 plasmid in PTPG produces the highest enhancement of coordinated neurovascular growth. Based on the results of in vitro experiments, we speculated that the REDV–IKVAV peptide provides the primary adhesion signal for ECs and nerve cells, while Talin1 amplifies integrin signaling. This concerted activation drives robust cellular migration and proliferation, culminating in neurovascular coupling. A capsaicin-induced sensory nerve dysfunction model was used to verify that the PTPG scaffold is the primary determinant of nerve regeneration (Supplementary Fig. 11). Compared with the PTPG group, CGRP and EMCN expression was significantly reduced in the capsaicin + PTPG group. No significant difference was detected between the capsaicin + PTPG group and the blank group, and the capsaicin group showed the lowest CGRP and EMCN levels among all groups. These findings indicate that scaffold-induced neuroangiogenesis is markedly attenuated in the absence of sensory nerves and is not solely attributable to the host’s intrinsic nerve repair capacity. Taken together, the data demonstrate that the PTPG scaffold promotes both angiogenesis and neurogenesis in a temporally coordinated manner, producing mature, functionally integrated neurovascular networks that enhance osteogenic activity.

The compelling preclinical results position the PTPG scaffold as a promising candidate for clinical translation. Its capacity to orchestrate neurovascular coupling and bone regeneration addresses a critical unmet need in repairing complex segmental defects. Advancing this technology will require rigorous safety and efficacy validation in large animal models, optimization of scalable manufacturing under Good Manufacturing Practice standards, and long-term evaluation of functional integration. Success in these endeavors could establish this bidirectional integrin activation strategy as a transformative approach in regenerative medicine.

### Single-cell profiling reveals PTPG scaffold-promoted neurovascularized bone regeneration via integrin signaling

To elucidate the cellular and molecular mechanisms underlying PTPG scaffold-mediated bone regeneration, we performed single-cell RNA sequencing on cells isolated from defect sites at 2 weeks post-implantation. After quality control, we retained 52,535 cells (PTPG: 21,254; control: 30,281). Unsupervised clustering and dimensionality reduction with Uniform Manifold Approximation and Projection/t-distributed Stochastic Neighbor Embedding (UMAP/t-SNE) identified nine major cell types involved in early repair: T cells, B cells, pericytes, osteoblasts, chondrocytes, ECs, neutrophils, SCs, and fibroblasts (Supplementary Fig. 12A–C).

Subclustering of the ECs revealed six subpopulations (Fig. 8C). The endo 2 subgroup of PTPG group showed significant upregulation of EMCN, a canonical marker of H-type ECs (H-ECs) (Fig. 8E), corroborating the immunohistochemical data. Pseudotime trajectory analysis indicated a progression toward H-ECs (Fig. 8D). The proliferation capacity of vascular ECs in bone marrow was mainly determined by H-ECs, which have a higher tendency to differentiate into arteries, forming the basis of local vascular network formation in bone tissue^54^. Kusumbe et al. demonstrated that vessel-related signaling in H-ECs plays a key role in mediating vascular formation and osteogenic coupling in bone reconstruction^55^. Kyoto Encyclopedia of Genes and Genomes (KEGG) pathway analysis and Gene Set Enrichment Analysis (GSEA) further indicated that the PTPG scaffold enhanced pathways associated with cytoskeletal organization, cell adhesion, VEGF signaling, and axon guidance (Fig. 8F, G and Supplementary Fig. 13A). Enrichment of integrin β1-mediated pathways, including those regulating tight junctions, the actin cytoskeleton, and adheren junctions, provides further evidence for ITGB1 activation by the PTPG scaffold. Furthermore, enrichment of axon guidance pathways suggests an unexpected role for ECs in orchestrating nerve regeneration, providing new mechanistic insight into the synergy of neurovascular repair. Western blot assay confirmed activation of the ITGB1/focal adhesion kinase/paxillin (ITGB1/FAK/PXN) axis (Supplementary Fig. 13B), supporting a mechanism by which PTPG scaffold promotes EC migration, vessel stability, and neurovascular interactions.

Likewise, subclustering of the SCs identified eight subclusters, with clusters 5, 7, and 8 representing a dedifferentiated, repair-promoting state (Fig. 9D, E). These subclusters displayed elevated myelin basic protein (MBP) expression (Fig. 9G). MBP promotes the formation and maturation of the myelin sheath by modulating SC signaling pathways and cytoskeletal organization, releasing signaling molecules that guide axonal growth^56^. Furthermore, myelinated SCs secrete neurotrophic factors and extracellular matrix components, which in turn, promote osteoblast generation and bone matrix deposition^42^.

Functional enrichment analysis revealed significant enrichment in pathways related to neural growth, vascular regeneration, and integrin-mediated signaling (Fig. 9F, H). These transcriptional changes are consistent with enhanced migratory, myelinating, and pro-angiogenic functions^57,58^. Activation of the ITGB1/FAK/PXN pathway was also evident in SCs (Supplementary Fig. 14), suggesting a shared mechanosensitive mechanism that facilitates both neural and vascular regeneration. Furthermore, based on neurovascular co-staining and functional analyses, we hypothesized that the PTPG scaffold promotes the formation of neurovascular units, coordinating vascular network development and neural innervation to facilitate high-quality bone regeneration.

Cell–cell communication analysis revealed enhanced signaling via fibronectin, pleiotrophin, angiopoietin-like proteins, and collagens in the PTPG group (Supplementary Fig. 15). The interaction between fibronectin and integrin supports adhesion and migration^59^, whereas pleiotrophin signaling promotes neural and vascular growth^60,61^. Collectively, these results suggest that PTPG scaffold establishes a pro-regenerative niche characterized by heightened metabolic activity, ECM remodeling, and coordinated multicellular crosstalk that synergistically accelerate bone defect repair.

This study presents a promising bifunctional strategy for bone regeneration. Nevertheless, several limitations should be acknowledged. Although the findings suggest that bidirectional ITGB1 activation may contribute to neurovascularized bone regeneration, the underlying regulatory network remains incompletely defined. Major downstream events within the ITGB1–FAK–PXN signaling axis have not been comprehensively validated, and mechanisms of intercellular communication were not examined. In addition, large-animal studies, such as rabbit or sheep models, were not performed to examine scaffold scalability and translational applicability. Future work should address these limitations through targeted mechanistic assays and validation in clinically relevant models.

In this study, we tested the hypothesis that concurrent activation of ITGB1 through both “outside-in” and “inside-out” signaling could synergistically promote neurovascular network formation and thereby influence osteogenesis in large bone defects. The PTPG scaffold, which integrates structural, biochemical, and genetic cues, was able to provide an environment that supported angiogenesis, neurogenesis, and osteogenesis in preclinical models in a spatially and temporally integrated manner. The results suggest that bidirectional activation of ITGB1 contributes to enhanced cellular migration, paracrine signaling, and coupling between vascular and neural components, which in turn may facilitate bone repair. Evidence from in vitro assays, in vivo models, and single-cell profiling supports the role of ITGB1-mediated signaling in orchestrating these processes. Overall, the work highlights a potential strategy for designing scaffolds that aim to coordinate neurovascular and osteogenic processes in bone regeneration. The study offers a venue for future translational exploration rather than definitive clinical conclusions.

## Methods

### Fabrication and characterization of PTPG scaffold

PLA (Aladdin, China, Mw ~ 60,000) scaffolds were fabricated by fused deposition modeling (FDM) 3D printing. The pore sizes were set at 400 μm in the outer region and 700 μm in the inner region. The printed scaffolds were subjected to alkaline treatment in dilute ammonia solution (pH 12.5) under magnetic stirring at room temperature for 6 h. The scaffolds were subsequently immersed in a 1% (w/v) HA (Aladdin, purity ≥97%) suspension in ethanol for 1 h and ultrasonicated in ethanol for 3 min to remove unbound HA, yielding PLA–HA scaffolds.

Talin1 plasmid DNA was available commercially (Genechem, Shanghai, China). Specifically, linearized vector was prepared by restriction digestion. The talin1 fragment was PCR-amplified using primers with 5′ homologous recombination sequences matching the vector ends. The linearized vector and PCR product were subjected to a recombination reaction for in vitro cyclization. The product was transformed, and single colonies were screened by PCR. Positive clones were verified by sequencing, and the correct plasmid was extracted for downstream use. The hyperbranched poly(β-amino ester)s (HPAEs) was obtained from an academic collaborator (Prof Zhou). The plasmid complex was prepared by mixing Talin1 plasmid with the HPAEs. Four chimeric peptides (LXW7–YIGSR, LXW7–IKVAV, REDV–YIGSR) were custom synthesized (Gill Biochemical, Shanghai, China). The peptide was prepared via solid-phase peptide synthesis. The first amino acid was covalently anchored to the resin, and subsequent amino acids were coupled sequentially from the C-terminus to the N-terminus. Each synthesis cycle consisted of deprotection and coupling steps, and the full-length peptide was finally cleaved from the resin.

The plasmid complex and peptides were incorporated into a 5% (w/v) GelMA solution by vortex mixing. The hydrogel mixture was then infused into PLA–HA scaffolds and crosslinked under ultraviolet light (365 nm) with an intensity of 10 mW/cm² for 60 s, resulting in the final peptide/Talin1 plasmid/PLA–HA/GelMA composite scaffold.

Field-emission scanning electron microscopy (FE-SEM): Scaffold specimens were frozen at −80 °C overnight and lyophilized using a vacuum freeze-dryer to remove residual water. The dried scaffolds were sputter-coated with a thin gold/palladium layer and imaged using FE-SEM (S-4800, Hitachi, Tokyo, Japan). Energy-dispersive X-ray spectroscopy (EDS, SDD550, IXRF Systems, Inc., Houston, TX, USA) was used to determine elemental composition of the PLA–HA scaffold. Hydrogel porosity was quantified from SEM images using ImageJ software (National Institute of Health, Bethesda, MD, USA).

Release curve: The PTPG scaffold was individually immersed in 1 mL of ion-free Tris-HCl buffer (pH 7.4) and maintained at 37 °C to simulate physiological conditions. At predetermined time-points (1, 3, 7, 14, 21, and 28 days), 400 μL of immersion medium was collected for analysis. Specimens were incubated with 0.5 mg/mL heparin solution at 37 °C for 1 h to dissociate Talin1 plasmid from the HPAEs. The concentration of DNA was then quantified using a UV spectrophotometer (Infinite M200 Pro, Tecan Group Ltd., Männedorf, Switzerland). Peptide release was measured using a microplate reader (Tecan) to provide quantitative assessment of peptide concentration in the collected specimens.

Circular dichroism (CD) spectroscopy: CD spectra of the original peptide and the peptide released from GelMA were measured using a CD spectropolarimeter in the wavelength range of 190–260 nm. The contents of secondary structures (α-helix, β-sheet, random coil, etc.) were calculated using the CDPro software package.

Agarose gel electrophoresis: Plasmid integrity and activity were verified using gel electrophoresis. Plasmids collected from the in vitro release supernatants were subjected to agarose gel electrophoresis, stained with ethidium bromide, and visualized under UV light.

Molecular docking: The three-dimensional structure of ITGB1 was predicted and optimized using AlphaFold (version 2.3.1). The protonation state of the REDV-IKVAV peptide was adjusted to simulate a pH of 7.4, and its three-dimensional structure was optimized and converted with Open Babel. Preprocessing of both ITGB1 and the REDV-IKVAV peptide, including the removal of water molecules, addition of hydrogen atoms, and assignment of partial charges, was carried out using AutoDock Tools (ADT3). The active binding pocket of ITGB1 was identified, and grid parameter files were prepared for subsequent docking simulations. Molecular docking was then performed using AutoDock Vina (version 1.2.0). Finally, the binding mode between the receptor and the ligand was visualized with PyMOL (version 2.5.0).

Reverse phase high performance liquid chromatography (RP-HPLC): Peptide characterization was performed using a Q-Exactive Plus Orbitrap Mass Spectrometer (Thermo Fisher Scientific, USA). The HPLC separation was conducted on a COSMOSIL Packed Column (4.6 × 250 mm, 5 μm) with a mobile phase of Solvent A (0.1% trifluoroacetic acid in 100% acetonitrile) and Solvent B (0.1% trifluoroacetic acid in 100% water), following a linear gradient of 13% A/87% B at 0.0 min, 38% A/62% B at 25.0 min, 100% A/0% B at 25.1 min, and stopping at 30.0 min, with a flow rate of 1.0 mL/min, injection volume of 20 μL, and UV detection at 220 nm.

Mass spectrometry analysis: MS analysis was carried out in positive electrospray ionization (ESI) mode with a scan range of *m*/*z* 300–2000, resolution of 70,000 (full scan), spray voltage of 3.5 kV, capillary temperature of 320 °C, sheath gas flow rate of 40 AU, auxiliary gas flow rate of 10 AU, and collision energy of 20–40 eV (for tandem MS if applicable), to verify the peptide’s molecular weight by matching the observed [M + H]⁺ ion peak with the theoretical value.

### In vitro study

The human umbilical vein endothelial cell line (HUVEC) and the rat Schwann cell line (RSC96) were obtained from the Stem Cell Bank of the Chinese Academy of Sciences (Shanghai, China). Two types of cells were cultured on four different scaffolds. PG = PLA-HA/GelMA, PPG = Peptide/PLA-HA/GelMA, TPG = Talin1 plasmid/PLA-HA/GelMA, PTPG = Peptide/Talin1 plasmid/PLA-HA/GelMA. Primary bone marrow-derived mesenchymal stem cells (BMSCs) were isolated from rats. The HUVEC and RSC96 cells were cultured in Dulbecco’s modified Eagle medium (DMEM; Gibco, Thermo Fisher Scientific, Waltham, MA, USA). The BMSCs were cultured in Minimum Essential Medium α (α-MEM; Gibco). All media were supplemented with 10% fetal bovine serum (FBS; InCellGene LLC, New York, USA) and 1% penicillin–streptomycin (InCellGene LLC, New York, USA). Cells were maintained at 37 °C in a humidified incubator with 5% CO₂ atmosphere.

The proliferative activity of HUVECs and RSC96 cells cultured on the scaffolds was quantitatively evaluated using the Cell Counting Kit-8 (CCK-8; Dojindo, Kumamoto, Japan). Cells were seeded onto the scaffolds. At predetermined time-points (1, 3, and 7 days), the culture medium was replaced with fresh medium containing CCK-8 reagent. After incubation at 37 °C for 2 h, 100 μL of supernatant from each sample was transferred to a 96-well plate to avoid interference from the scaffold material. The absorbance of the formazan product, which correlates with cell viability, was measured at 450 nm using a microplate reader (BioTek, Winooski, VT, USA).

Cell viability was evaluated using the LIVE/DEAD™ cell viability assay (Thermo Fisher Scientific) after 1, 3, and 7 days of culture on the scaffolds. Scaffolds were stained with a mixture containing calcein AM (0.5 μL/mL) and ethidium homodimer-1 (2 μL/mL) at room temperature for 1 h. Specimens were imaged using a confocal laser scanning microscope (CLSM; Nikon Corp., Tokyo, Japan).

A wound healing assay was used to evaluate cellular migration capacity. Approximately 5 × 10⁴ cells were seeded into 24-well plates. A uniform scratch was created in the cell monolayer using a sterile pipette tip. After 24 h of incubation, specimens were washed three times with phosphate-buffered saline (PBS) and fixed with 4% paraformaldehyde for 20 min at room temperature. Fluorescence images of the wound area were acquired using confocal laser scanning microscopy (CLSM; Leica Microsystems, Wetzlar, Germany). Migration rates (%) were calculated as (*A₀* − *A*_*n*_)/*A₀* × 100%, where *A₀* = the initial wound area at time 0 (i.e., immediately after the scratch is made), and *A*_*n*_ = the wound area remaining after incubation (e.g., at 24 h).

Matrigel® (Corning Inc., Corning, NY, USA) was thawed on ice and 24-well plates were coated with 200 μL per well. The gel was polymerized at 37 °C for 60 min. Approximately 2 × 10⁴ endothelial cells were seeded onto the Matrigel® surface in medium containing scaffold extracts. Tubular structures were imaged using CLSM after a 6 h incubation period. Quantitative analysis of angiogenesis was performed with ImageJ software using the Angiogenesis Analyzer plugin. Total tube length, branch points, and nodes were measured in 3–4 representative fields per condition, and mean values were reported.

Gene expression in HUVECs and RSC96 cells was evaluated with quantitative real-time polymerase chain reaction (qRT-PCR) after 7 days of culture. Total RNA was extracted with TRIzol reagent (Invitrogen, Thermo Fisher Scientific), and purity was determined by absorbance at 260/280 nm. Complementary DNA (cDNA) was synthesized using the PrimeScript RT reagent kit (TaKaRa, Kusatsu, Japan). Reaction was performed with the ABI Prism 7500 Real-Time PCR System (Applied Biosystems, Foster City, CA, USA). Relative expression levels were calculated using the 2^ΔΔCT^ method. Primer sequences are listed in supplementary Table 1.

The ITGB1 siRNA and non-targeting negative control (NC) were designed and synthesized by Gene Create Co., Ltd. (Wuhan, China). Transfections were carried out using Lipofectamine 3000 (Invitrogen, USA) following the manufacturer’s instructions with minor modifications. Specifically, 5 μL of 20 μM ITGB1 siRNA (or NC) was diluted in 125 μL Opti-MEM Reduced Serum Medium and mixed with an equal volume of Lipofectamine 3000 diluted in Opti-MEM after 5 min. The siRNA-lipid complexes were incubated at room temperature for 15 min and then added dropwise to cells in 6-well plates at 60–70% confluency in antibiotic-free complete medium. Cells were incubated at 37 °C in 5% CO₂ for 6 h, after which the medium was replaced with fresh complete medium supplemented with 10% fetal bovine serum and 1% penicillin-streptomycin.

The cells were fixed with 4% paraformaldehyde 7 days after seeding. After fixation, the cells were permeabilized with 0.1% Triton X-100, and stained with phalloidin to visualize cytoskeletal organization. Specimens were imaged using the Z-stack tomography function of the CLSM. Three-dimensional reconstructions were generated to evaluate cell penetration and migration distance toward the scaffold interior.

The hyperbranched polymer was mixed with Talin1 plasmid DNA at a mass ratio of 30:1. The resulting complexes were stabilized in PBS for 25 min before use. The stabilized aggregates were then added to the cell culture medium and incubated with cells for 24 h. Confocal laser scanning microscopy (Leica Microsystems) was used after incubation to observe and quantify fluorescence intensity as an indicator of plasmid transfection efficacy.

The cells were fixed with 4% paraformaldehyde and washed three times with PBS after 7 and 14 days of osteogenic induction. They were then stained with an alkaline phosphatase (ALP) color development kit (Beyotime Biotechnology, Shanghai, China) following the manufacturer’s instructions. Images were captured using an inverted microscope (Leica Microsystems, Wetzlar, Germany). For quantitative evaluation, osteoblasts were lysed with 1% Triton X-100, and absorbance at 405 nm was measured using the ALP assay kit. Values were normalized to total protein content.

After 7 and 14 days of osteogenic induction, the cells were fixed with 4% paraformaldehyde, washed with PBS, and stained with Alizarin Red S solution (Solarbio, Beijing, China; pH 4.2) for 10 min. Specimens were washed with deionized water and examined under the Leica inverted microscope. For quantification, the bound dye was solubilized in 10% cetylpyridinium chloride for 1 h. Absorbance was measured at 562 nm using the Biotek microplate reader.

Protein expression in HUVECs and RSC96 cells was analyzed with western blotting after 7 days of culture. The cells were lysed in buffer containing 1× phosphatase inhibitor. Total protein concentration was determined using the Pierce BCA protein assay kit (CWBIO, Beijing, China). Protein samples were separated on 10% SDS–polyacrylamide gels and transferred to polyvinylidene difluoride membranes (EMD Millipore Corp., Burlington, MA, USA). Membranes were blocked with protein-free rapid blocking buffer (EpiZyme Biotechnology, Shanghai, China) and incubated with primary antibodies against integrin β1 (ITGB1; 1:1000), nerve growth factor (NGF; 1:1000), brain-derived neurotrophic factor (BDNF; 1:1000), hypoxia-inducible factor-1α (HIF-1α; 1:1000), vascular endothelial growth factor (VEGF; 1:1000), Runt-related transcription factor 2 (RUNX2; 1:1000), bone morphogenetic protein-2 (BMP2; 1:1000), and osteocalcin (OCN; 1:1000) (all from Santa Cruz Biotechnology, Dallas, TX, USA). After overnight incubation at 4 °C, membranes were probed with horse radish peroxidase-conjugated secondary antibodies (Affinity Biosciences, Cincinnati, OH, USA). Bands were visualized using the ChemiDoc imaging system (Bio-Rad Laboratories, Hercules, CA, USA) and quantified with ImageJ software. β-actin was used as the loading control.

### In vivo study

Forty-two six-week-old male Sprague–Dawley rats were randomly divided into four groups (*n* = 6 independent replicates). A femoral bone defect model was established to evaluate the osteogenic capacity of the scaffolds. All animal experiments were conducted in accordance with the guidelines of Application for Laboratory Animal Welfare and Ethical review, School of Stomatology, Air Force Military Medical University, with the corresponding approval number: 2025 kq-006. The rats were anesthetized with 3% pentobarbital sodium. A cylindrical defect (3.0 mm in diameter × 3.0 mm in depth) was created in the femur using an electric saw. Physiological saline was used to cool the site and reduce thermal damage. A cylindrical scaffold of the corresponding dimensions was implanted into each defect site. Each rat received 25,000 U of penicillin by intramuscular injection daily for three consecutive days after implantation. The rats were housed under specific pathogen-free conditions with a 12 h light/dark cycle. The rats were euthanized 4 and 8 weeks after surgery with an overdose of pentobarbital sodium. The femurs were harvested for analysis.

Harvested bone specimens were scanned using micro-computed tomography (micro-CT; Leica Microsystems, Wetzlar, Germany) at 18 μm resolution. Three-dimensional reconstructions were generated using the Inveon Research Workplace software (Siemens Medical Solutions USA, Inc., Malvern, PA, USA). A cylindrical region of interest was defined at the defect site, and new bone volume was quantified. Bone volume/total volume ratio (BV/TV) and trabecular number (Tb.N) were analyzed using the Inveon Research Acquisition software (Siemens). Frozen sections were prepared by embedding femurs in optimal cutting temperature (OCT) compound after fixation and EDTA decalcification. Sections were permeabilized with 0.3% Triton X-100 for 20 min, blocked with 10% goat serum for 30 min, and incubated with primary antibodies overnight at 4 °C. Sections were incubated the following day with fluorescent secondary antibodies for 1 h at room temperature. Nuclei were counterstained with 4′,6-diamidino-2-phenylindole (DAPI) for 10 min, and sections were mounted with antifade reagent. Fluorescence imaging was performed with the Leica CLSM. Semi-quantitative analysis of fluorescence intensity was performed using ImageJ software.

The compressive strength and elastic modulus of the scaffolds were measured using a universal material testing machine (Instron Corp., Norwood, MA, USA). Each specimen was positioned at the center of the compression fixture, and the crosshead speed was set at 1 mm/min. Force–displacement curves were recorded until specimen failure. Compressive strength was calculated by dividing the maximum load by the cross-sectional area of the specimen. Elastic modulus was derived from the slope of the linear region of the force–displacement curve. All tests were performed at room temperature. Five specimens were tested in each group, and mean values were reported.

Male SD rats aged 2 months were selected as experimental animals. Under sterile conditions, the dorsal muscles were dissected, and the ligaments of the vertebral articular surfaces were transected to expose the L3-L5 lateral processes, with the dorsal root ganglia (DRG) visualized. A capsaicin solution was directly injected into the DRG tissue for three consecutive days, with dosages increased incrementally: 30 mg/kg on Day 1, 50 mg/kg on Day 2, and 70 mg/kg on Day 3. After establishing the sensory nerve denervation model, the PTPG scaffold was implanted, and the experimental groups were designated as follows: Blank group, PTPG group, Capsaicin group, and Capsaicin + PTPG group.

Femurs were fixed in 4% neutral-buffered formaldehyde and decalcified in 10% ethylenediamine tetra-acetic acid (EDTA). After dehydration with a graded ethanol series and xylene clearing, the demineralized femur specimens were embedded in paraffin and sectioned at a thickness of 4 μm. Sections were mounted on charged glass slides for subsequent staining. For Masson trichrome staining, nuclei were stained with Weigert’s iron hematoxylin, collagen fibrils with Biebrich scarlet–acid fuchsin, and color differentiation achieved with phosphotungstic–phosphomolybdic acid. For hematoxylin and eosin staining, nuclei were stained with hematoxylin and cytoplasm with eosin. For immunohistochemistry, sections were deparaffinized, antigen retrieval was performed, and endogenous peroxidase was quenched with 3% hydrogen peroxide. Sections were incubated with primary antibodies overnight at 4 °C, followed by incubation with biotinylated secondary antibodies and horseradish peroxidase–streptavidin complex. Diaminobenzidine was used for visualization, and nuclei were counterstained with hematoxylin.

Bone defect tissues from blank and PTPG scaffold groups (*n* = 6 per group) were harvested for single-cell ribonucleic acid sequencing (scRNA-seq). The specimens were minced into 1–3 mm³ fragments after removal of the surrounding muscle and connective tissues. The minced tissues were digested with type I and type IV collagenase, red blood cells were lysed, and suspensions were filtered through 70 μm and 40 μm cell strainers to obtain single-cell suspensions. Red blood cells and immune cells were depleted using antibodies against HIS49 (erythrocytes) and CD45 (immune cells) via flow cytometry. Cell viability (>90%) was confirmed with an automated cell counter. Single-cell barcoding and library preparation were performed using the Chromium platform (10x Genomics, Pleasanton, CA, USA). Gel bead-in-emulsions (GEMs) were generated with microfluidic chips, and reverse transcription was performed to capture and convert messenger RNA into cDNA. Libraries were prepared and quality-checked using a Qubit Fluorometer (Thermo Fisher Scientific) and an Agilent 2100 Bioanalyzer (Agilent Technologies, Santa Clara, CA, USA). Libraries meeting concentration and size criteria were sequenced on an Illumina NovaSeq platform (Illumina Inc., San Diego, CA, USA).

### Statistical analyses

All data were presented as means ± standard deviations. Statistical analysis was performed using SPSS software, version 23.0 (IBM Corp., Armonk, NY, USA). All key findings were reproduced in at least three independent experiments. One-way analysis of variance (ANOVA) was used to analyze differences among groups. A value of *P* < 0.05 was considered statistically significant, with *P* < 0.01 and *P* < 0.001 indicating higher levels of significance.

### Reporting summary

Further information on research design is available in the Nature Portfolio Reporting Summary linked to this article.

## Supplementary information

Supplementary information

Reporting Summary

Transparent Peer Review file

## Source data

Source Data

## Data Availability

All data supporting the findings of this study are available within the article and its supplementary files. The scRNA-seq data generated in this study have been deposited in the SRA data base under accession code PRJNA1434018. Source data is available for Figs. 2–8 and Supplementary Figs. 2–14 in the associated source data file. Source data are provided with this paper.
