# Supplementary material for: Bidirectional integrin β1 activation synergizes neurovascular coupling and enhances bone regeneration
Source: Nat Commun. 2026 Jun 12;17:7610. doi: 10.1038/s41467-026-74071-3 (PMC13424096; doi:10.1038/s41467-026-74071-3)
Supplement: Supplementary file 1 — Supplementary information [file 41467_2026_74071_MOESM1_ESM.pdf]

Supplementary Information

Bidirectional integrin β1 activation synergizes neurovascular coupling and enhances bone regeneration

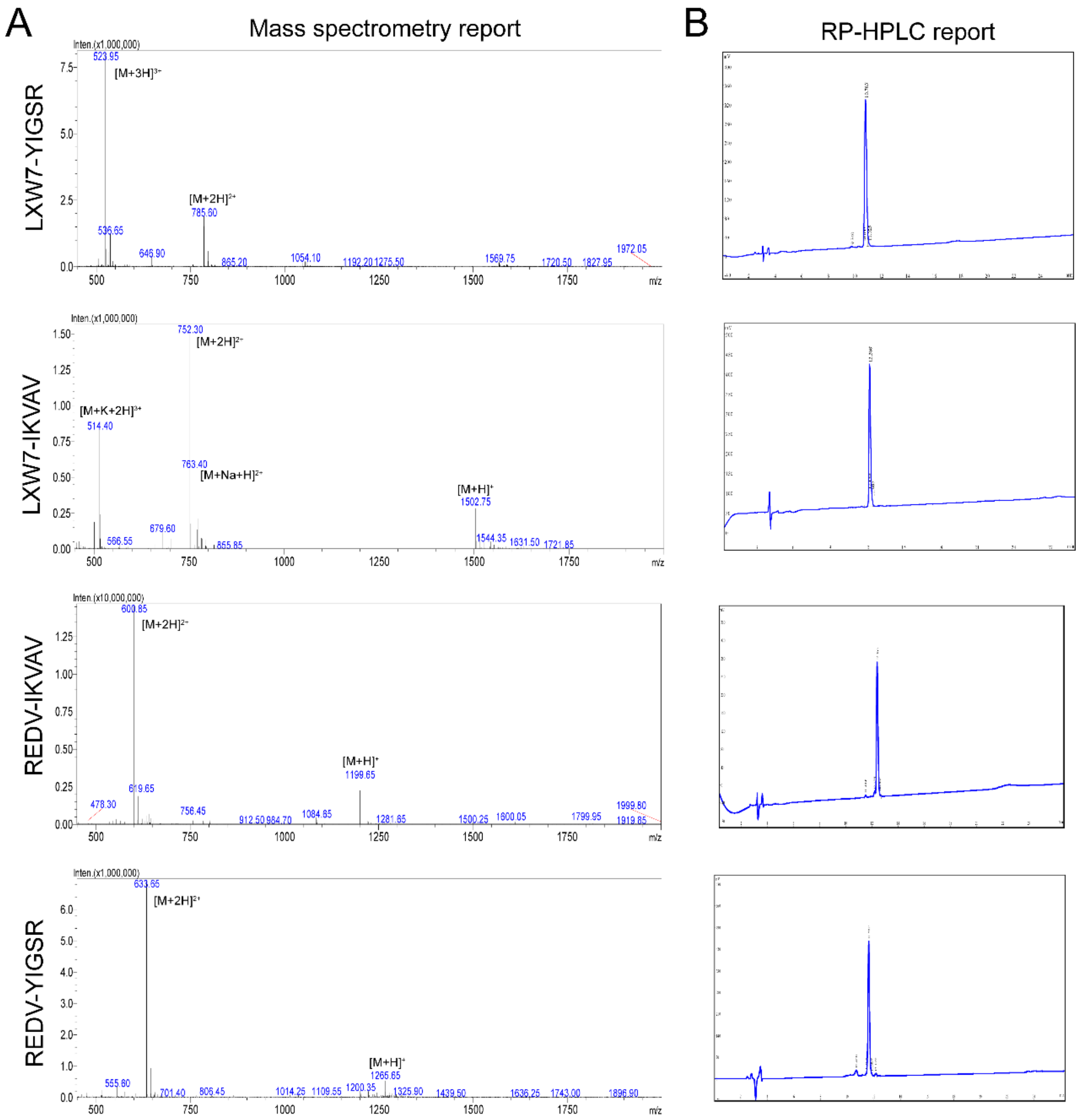

Figure S1. Chemical characterization of the synthesized chimeric peptides.

(A) Mass spectrometry (MS) spectrum confirming the molecular weight. (B) Reverse-phase high-performance liquid chromatography (RP-HPLC) profile showing peptide purity (>95%).

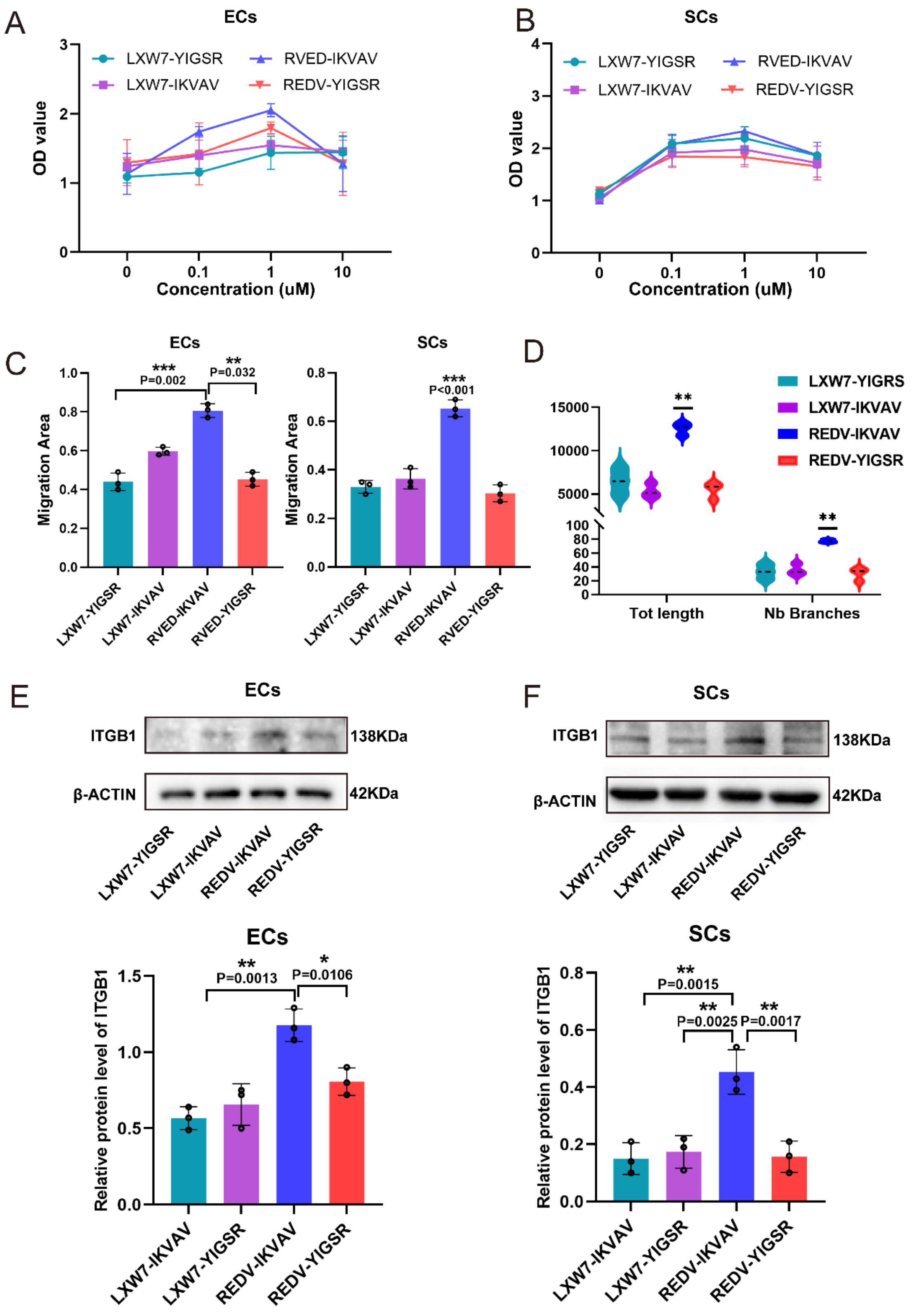

Figure S2. Effects of synthesized chimeric peptides on endothelial and Schwann cell functions.

(A-B) CCK-8 assay of ECs (A) and SCs (B) treated with four synthesized chimeric peptides for 1, 3, and 7 days (n = 3 independent replicates); (C) Migration area analysis

of ECs and SCs treated with four synthesized chimeric peptides (**$P < 0.01$, ***$P < 0.001$, n = 3 independent replicates); (D) Statistical analysis of total length (tot length) and number of branches (Nb branches) of tube formation (**$P < 0.01$, n = 3 independent replicates); (E) Western blot and quantitative analyses of ITGB1 expression in ECs treated with the indicated peptides (*$P < 0.05$, **$P < 0.01$, n = 3 independent replicates); (F) Western blot and quantitative analyses of ITGB1 expression in SCs treated with the indicated peptides (**$P < 0.01$, n = 3 independent replicates). Data is represented as the mean ± SD. The P value of statistical significance is determined by one-way ANOVA with Tukey's post-hoc test. LXW7–YIGSR (Cyclo-Arg-Gly-Asp-Gly-Gly-Gly-Tyr-Ile-Gly-Ser-Arg), LXW7–IKVAV (Cyclo-Arg-Gly-Asp-Gly-Gly-Gly-Ile-Lys-Val-Ala-Val), REDV–IKVAV (Arg-Glu-Asp-Val-Gly-Gly-Gly-Ile-Lys-Val-Ala-Val), REDV–YIGSR (Arg-Glu-Asp-Val-Gly-Gly-Gly-Tyr-Ile-Gly-Ser-Arg).

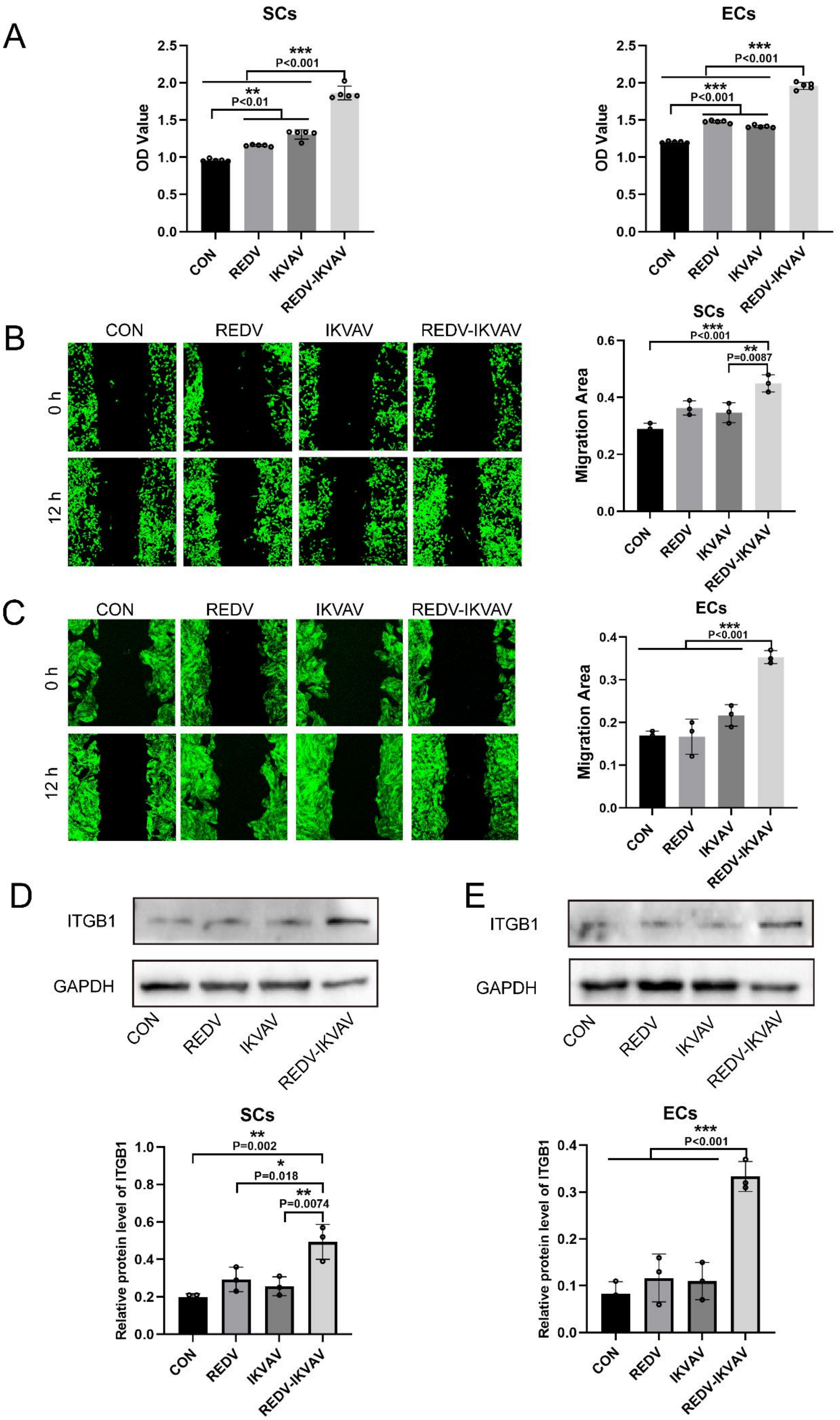
A
SCs
ECs
OD Value
***
P<0.001
**
P<0.01
CON
REDV
IKVAV
REDV-IKVAV
B
0 h
12 h
Migration Area
P=0.0087
C
D
E
ITGB1
GAPDH
Relative protein level of ITGB1
P=0.002
*
P=0.018
P=0.0074

Figure S3. Comparative effects of REDV, IKVAV and REDV–IKVAV peptides on neurovascular cells.

(A) CCK-8 assay of SCs and ECs treated with REDV, IKVAV, and REDV–IKVAV peptides for 1, 3, and 7 days (**$P < 0.01$, ***$P < 0.001$, $n = 3$ independent replicates); (B-C) Migration area analysis of SCs (B) and ECs (C) treated with REDV, IKVAV, and REDV–IKVAV peptides (***$P < 0.001$, $n = 3$ independent replicates); (D) Western blot and quantitative analyses of ITGB1 expression in SCs treated with REDV, IKVAV, and REDV–IKVAV peptides (*$P < 0.05$, **$P < 0.01$, $n = 3$ independent replicates); (E) Western blot and quantitative analyses of ITGB1 expression in ECs treated with REDV, IKVAV, and REDV–IKVAV peptides (***$P < 0.001$, $n = 3$ independent replicates). Data is represented as the mean ± SD. The P value of statistical significance is determined by two tailed one-way ANOVA with Tukey's post-hoc test.

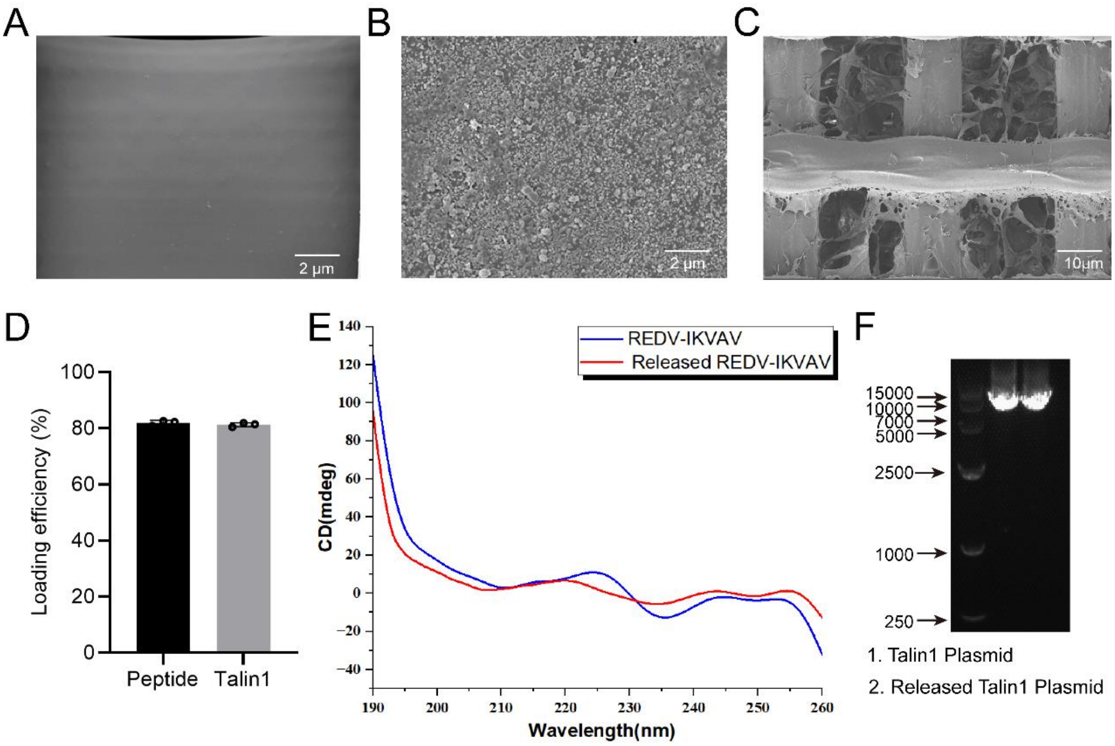

Figure S4. Characterization of PTPG scaffold.

(A) Surface morphology of PLA scaffold (scale bar = 2 μm); (B) Surface morphology of PLA-HA scaffold (scale bar = 2 μm); (C) Surface morphology of inner PTPG scaffold (scale bar = 10 μm); (D) Loading efficiency of peptide and talin1 plasmid; (E) Circular Dichroism (CD) analysis of native REDV–IKVAV and that released from the hydrogel; (F) Gel electrophoresis analysis of native Talin1 plasmid and that released from the hydrogel.

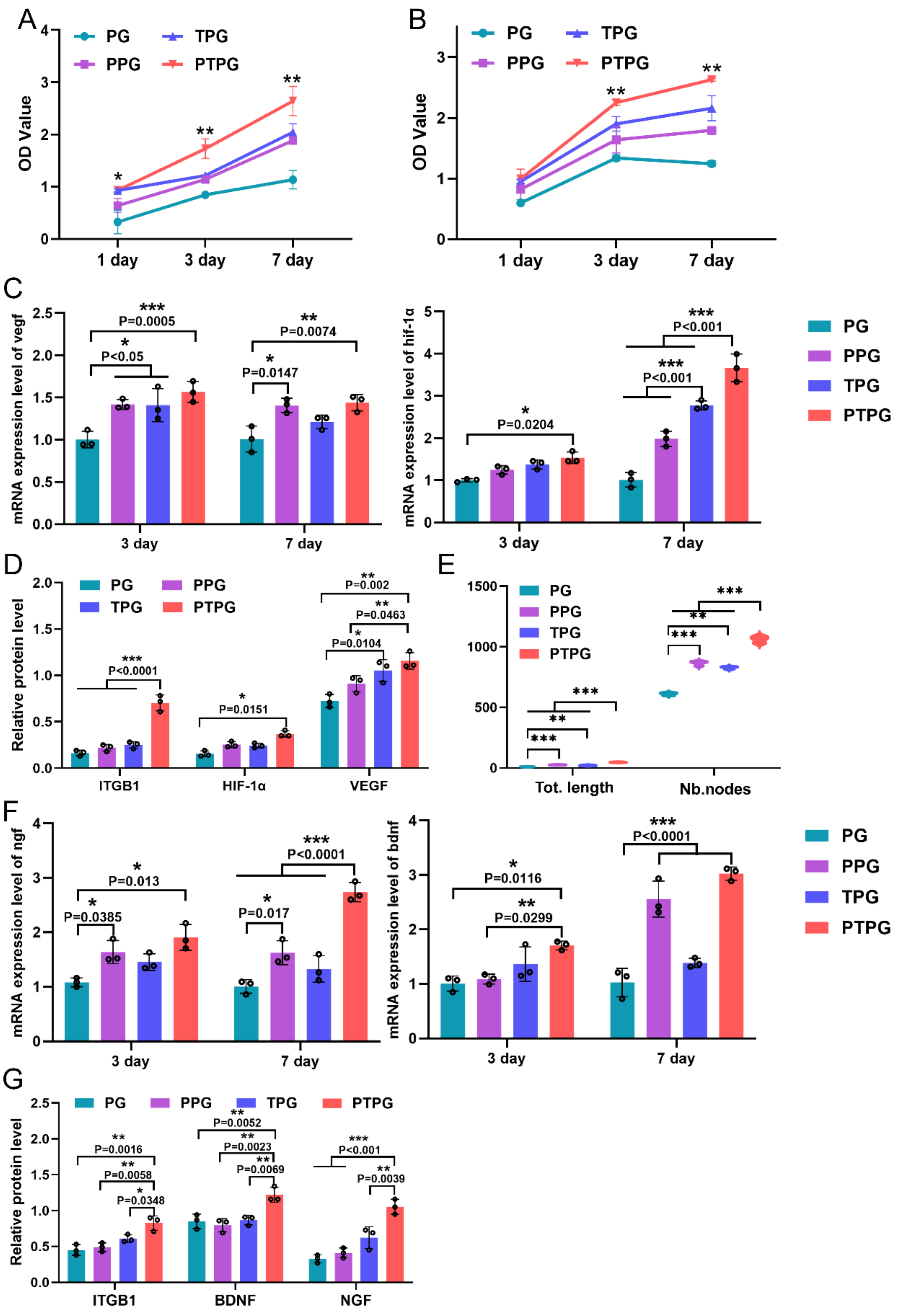

Figure S5. PTPG scaffold enhances neurovascular cell function in vitro.

(A-B) CCK-8 analysis of ECs (A) and SCs (B) cultured on different scaffolds for 1, 3, and 7 days (*P < 0.05, **P < 0.01, n = 3 independent replicates); (C) mRNA expression of Vegf and Hif-1α were evaluated by qRT-PCR in ECs (*P < 0.05, **P < 0.01,***P <

0.001, n = 3 independent replicates); (D) Quantitative analyses of ITGB1, VEGF, and HIF-1α expression in ECs (*P < 0.05, **P < 0.01,***P < 0.001, n = 3 independent replicates); (E) Statistical analysis of total length and number of nodes of tube formation (**P < 0.01, ***P < 0.001, n = 3 independent replicates); (F) mRNA expression of Ngf and Bdnf were evaluated by qRT-PCR in SCs (*P < 0.05, **P < 0.01,***P < 0.001, n = 3 independent replicates); (G) Quantitative analyses of NGF, BDNF, and ITGB1 expression in SCs (*P < 0.05, **P < 0.01, ***P < 0.001, n = 3 independent replicates). Data is represented as the mean ± SD. The P value of statistical significance is determined by one-way ANOVA with Tukey's post-hoc test. The experiment was divided into four groups: PG, PLA-HA/GelMA; PPG, Peptide/PLA-HA/GelMA; TPG, Talin1 plasmid/PLA-HA/GelMA; PTPG, Peptide/Talin1 plasmid/PLA-HA/GelMA.

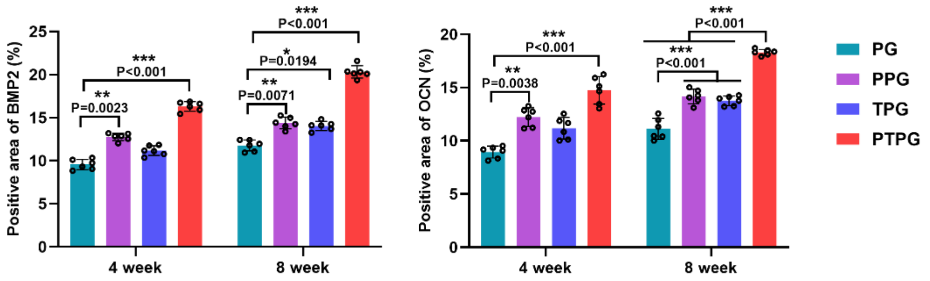

Figure S6. Quantification of BMP2 and OCN immunohistochemical staining.

Quantitative analysis of the expression of BMP2 and OCN in the defect area. (*P < 0.05, **P < 0.01, ***P < 0.001, n = 6 independent replicates). Data is represented as the mean ± SD. The P value of statistical significance is determined by one-way ANOVA with Tukey's post-hoc test. The experiment was divided into four groups: PG, PLA-HA/GelMA; PPG, Peptide/PLA-HA/GelMA; TPG, Talin1 plasmid/PLA-HA/GelMA; PTPG, Peptide/Talin1 plasmid/PLA-HA/GelMA.

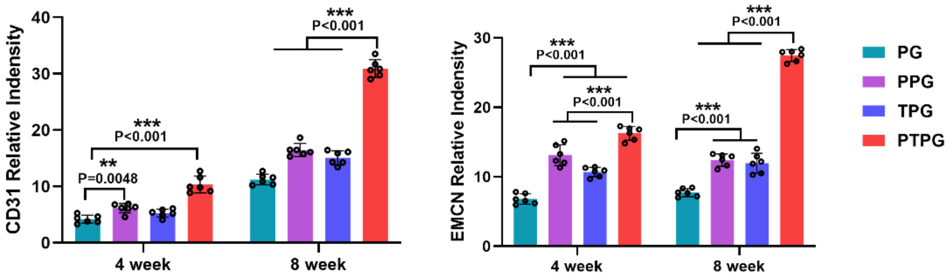

Figure S7. Quantitative analysis of immunofluorescence staining of CD31 and EMCN.

Immunofluorescence intensity analysis of CD31 and EMCN in the defect area at 4 and 8 weeks post-implantation. (**P < 0.01, ***P < 0.001 n = 6 independent replicates). Data is represented as the mean ± SD. The P value of statistical significance is determined by one-way ANOVA with Tukey's post-hoc test. The experiment was divided into four groups: PG, PLA-HA/GelMA; PPG, Peptide/PLA-HA/GelMA; TPG, Talin1 plasmid/PLA-HA/GelMA; PTPG, Peptide/Talin1 plasmid/PLA-HA/GelMA.

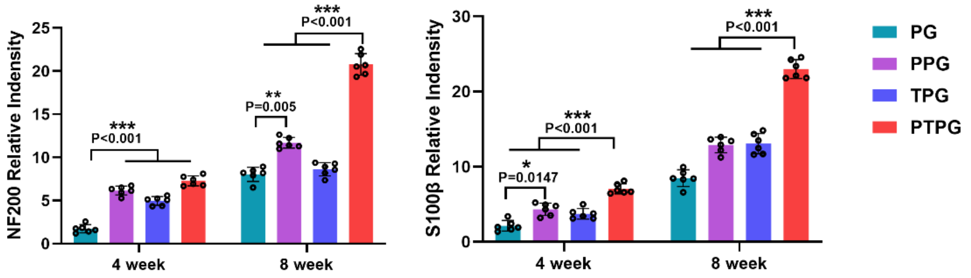

Figure S8. Quantitative analysis of immunofluorescence staining of NF200 and S100β.

Immunofluorescence intensity analysis of NF200 and S100β in the defect area at 4 and 8 weeks post-implantation. (*P < 0.05, **P < 0.01, ***P < 0.001, n = 6 independent replicates). Data is represented as the mean ± SD. The P value of statistical significance is determined by one-way ANOVA with Tukey's post-hoc test. The experiment was divided into four groups: PG, PLA-HA/GelMA; PPG, Peptide/PLA-HA/GelMA; TPG, Talin1 plasmid/PLA-HA/GelMA; PTPG, Peptide/Talin1 plasmid/PLA-HA/GelMA.

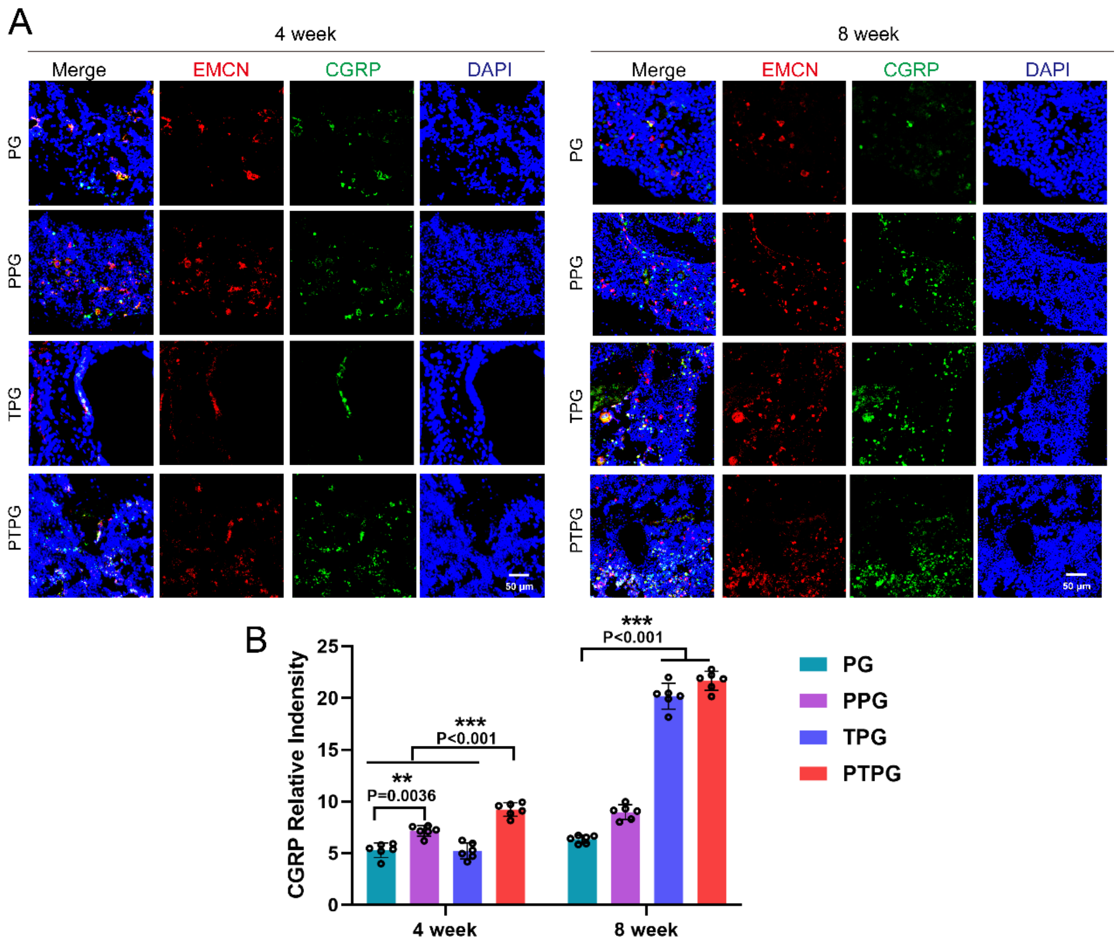

Figure S9. PTPG scaffold promotes the regeneration of sensory nerves in vivo.

(A) Representative immunofluorescence images of EMCN and CGRP in the defect area at 4 and 8 weeks post-implantation; (B) Immunofluorescence intensity analysis of CGRP (**$P < 0.01$, ***$P < 0.001$, $n = 6$ independent replicates). Data is represented as the mean ± SD. The P value of statistical significance is determined by one-way ANOVA with Tukey's post-hoc test. The experiment was divided into four groups: PG, PLA-HA/GelMA; PPG, Peptide/PLA-HA/GelMA; TPG, Talin1 plasmid/PLA-HA/GelMA; PTPG, Peptide/Talin1 plasmid/PLA-HA/GelMA.

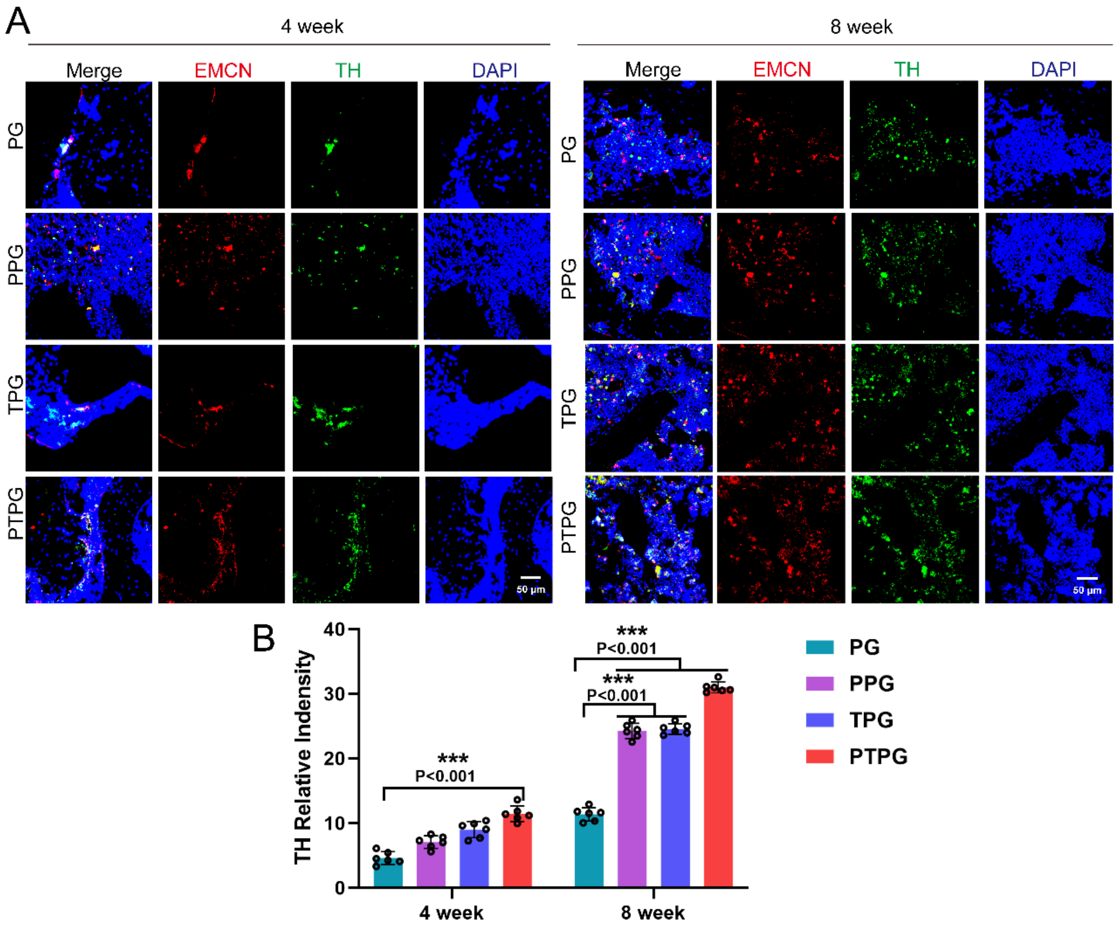

Figure S10. PTPG scaffold promotes the regeneration of sympathetic nerves in vivo.

(A) Representative immunofluorescence images of EMCN and TH in the defect area at 4 and 8 weeks post-implantation; (B) Immunofluorescence intensity analysis of TH (***$P < 0.001$, $n = 6$ independent replicates). Data is represented as the mean ± SD. The P value of statistical significance is determined by one-way ANOVA with Tukey's post-hoc test. The experiment was divided into four groups: PG, PLA-HA/GelMA; PPG, Peptide/PLA-HA/GelMA; TPG, Talin1 plasmid/PLA-HA/GelMA; PTPG, Peptide/Talin1 plasmid/PLA-HA/GelMA.

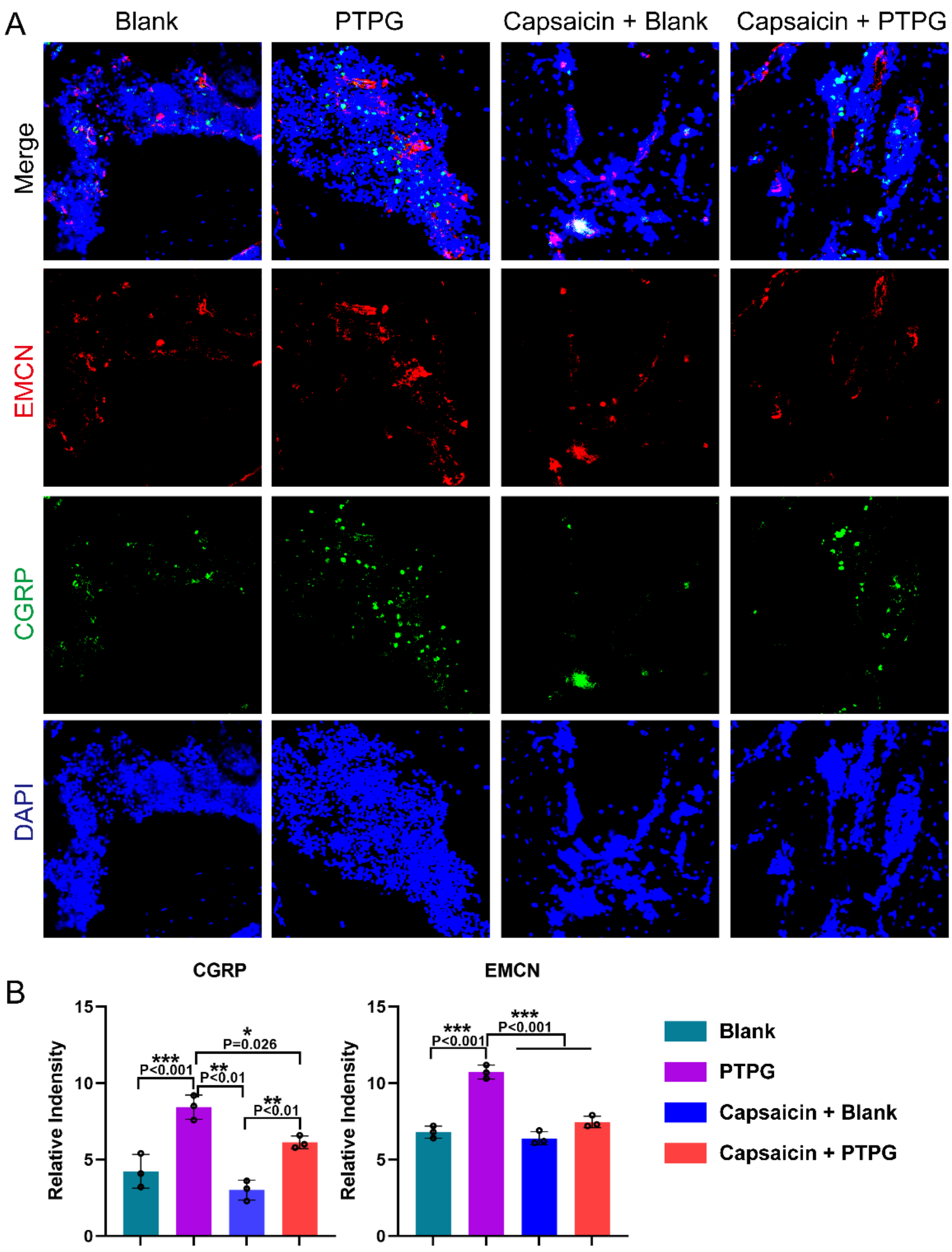

Figure S11. Immunofluorescence staining of EMCN and CGRP in a sensory nerve dysfunction model at 4 weeks post-implantation

(A) Representative immunofluorescence images of EMCN and CGRP at 4 weeks. (B) Immunofluorescence intensity analysis of EMCN and CGRP (*$P < 0.05$, **$P < 0.01$, ***$P < 0.001$, $n = 3$ independent replicates). Data is represented as the mean ± SD. The P value of statistical significance is determined by one-way ANOVA with Tukey's post-hoc test. PTPG, Peptide/Talin1 plasmid/PLA-HA/GelMA.

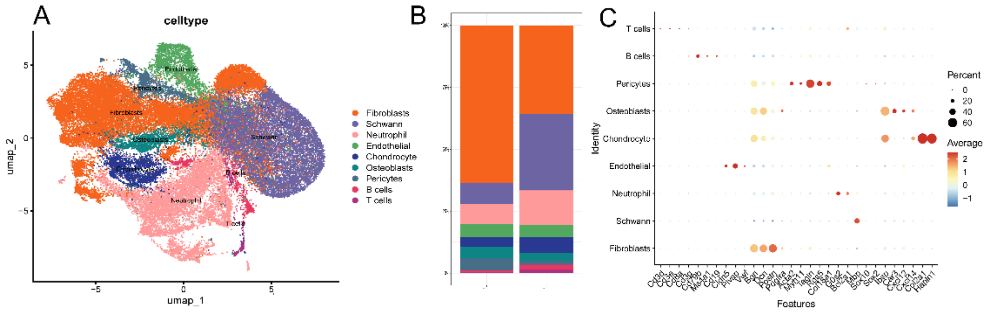

Figure S12. Single-cell transcriptomic profiling of cells within the bone defect microenvironment at 2 weeks post-implantation.

(A) Uniform Manifold Approximation and Projection (UMAP) plot of 52,535 high-quality cells isolated from the defect site, colored by cell type identity. Nine major cell clusters were identified based on canonical marker gene expression: T cells (Cd3d), B cells (Cd79a), Pericytes (Rgs5, Cspg4), Osteoblasts (Bglap, Sp7), Chondrocytes (Col2a1, Acan), Endothelial cells (Pecam1, Cdh5), Neutrophils (S100a8, S100a9), Schwann cells (Plp1, Mpz), and Fibroblasts (Col1a1, Dcn); (B) Stacked bar plot showing the proportional composition of each cell type across the two experimental groups. (C) Dot plot visualizing the expression (scaled average expression and percentage of cells expressing) of top marker genes used to define each cell cluster.

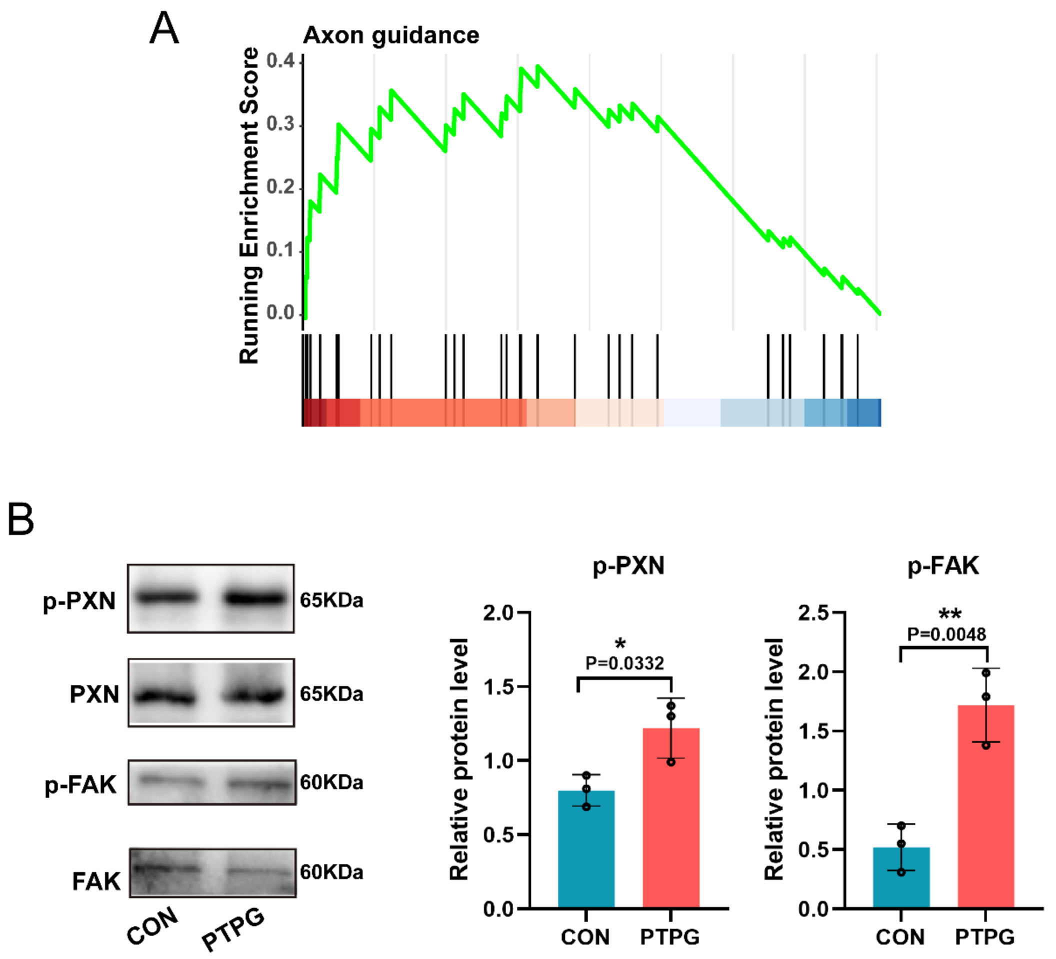

Figure S13. Pathway analysis and validation of the EC subpopulation.

(A) GSEA of axon guidance signaling. (B) Western blot (left) and quantitative analyses (right) of key proteins in the ITGB1 downstream signaling pathway in ECs cultured in the control (CON) or PTPG group for 7 days. (*$P < 0.05$, **$P < 0.01$, $n = 3$ independent replicates). Data is represented as the mean ± SD. The P value of statistical significance is determined by two tailed Student's t-test. PTPG, Peptide/Talin1 plasmid/PLA-HA/GelMA.

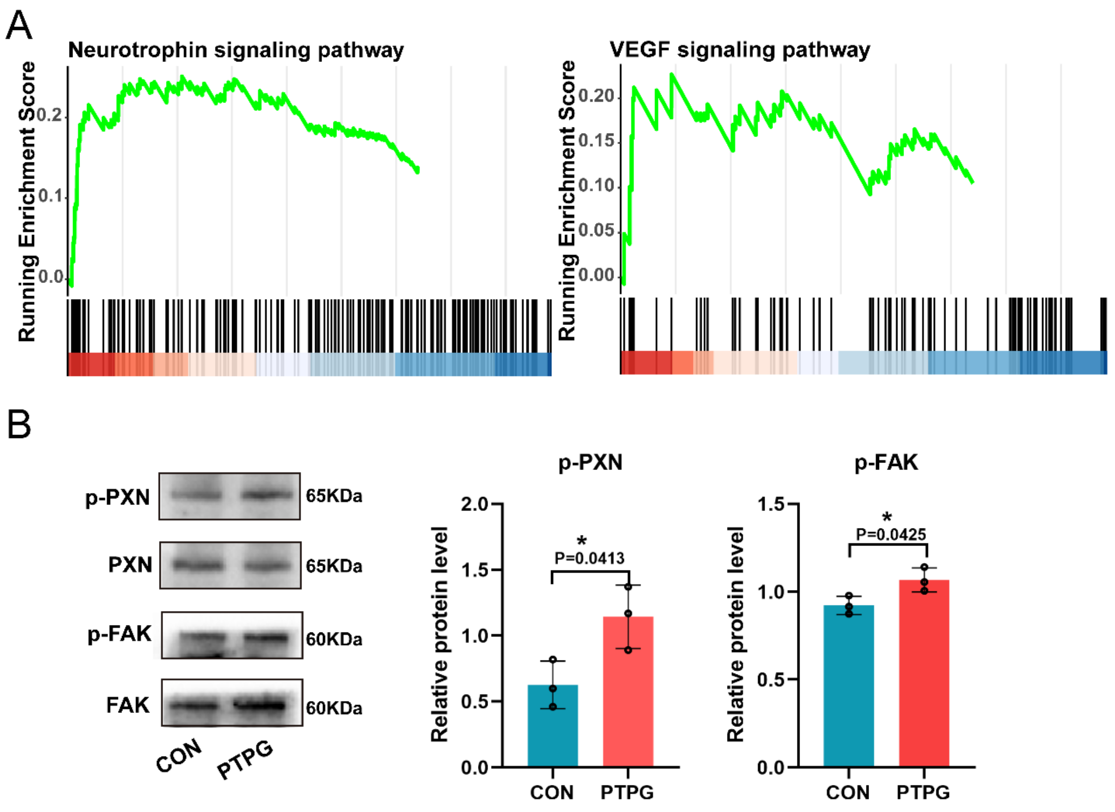

Figure S14. Pathway analysis and validation of the SC subpopulation.

(A) GSEA of neurotrophin signaling pathway and VEGF signaling pathway; (B) Western blot (left) and quantitative analyses (right) of key proteins in the integrin β1 (ITGB1) downstream signaling pathway in SCs cultured in the control (CON) or PTPG group for 7 days. (*$P < 0.05$, $n = 3$ independent replicates). Data is represented as the mean ± SD. The P value of statistical significance is determined by two tailed Student's t-test. PTPG, Peptide/Talin1 plasmid/PLA-HA/GelMA.

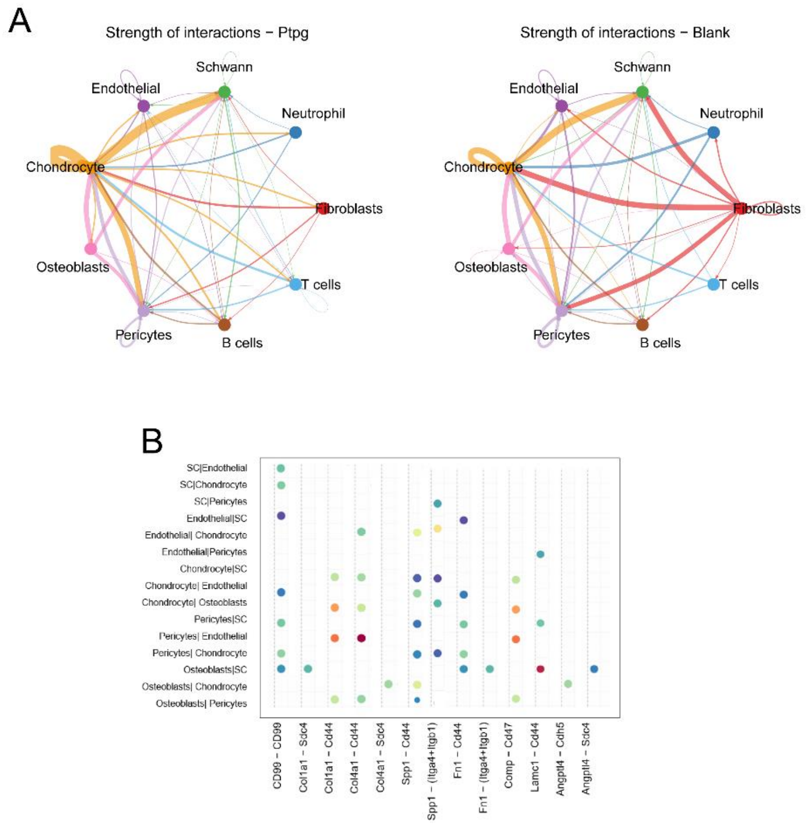

Figure S15. Analysis of intercellular communication networks within the bone defect microenvironment.

(A) Chord diagram visualizing the strength and direction of ligand-receptor mediated communication among the nine major cell types identified by scRNA-seq. Cell types are represented as nodes around the circle; connecting chords represent inferred interactions, with chord thickness proportional to the communication probability. The network was inferred from scRNA-seq data using the CellChat computational toolkit; (B) Bubble plot summarizing the number of significant ligand-receptor interactions between sender and receiver cell clusters. The size of each circle corresponds to the number of unique interactions, highlighting predominant communication pathways such as VEGF, collagen, and fibronectin signaling. This analysis reveals enhanced multicellular crosstalk in the PTPG group, suggesting a more coordinated pro-regenerative microenvironment.

Table S1. Primer sequences for qRT-PCR analysis

Primer	Sequences
Gapdh	ACAGCAACAGGGTGGTGGAC
	TTTGAGGGTGCAGCGAACTT
Vegf	CAGGAGTACCCCGACGAGATAG
	TGTGCTGGCTTTGGTGAGGT
Hif-1α	TCGAAGTAGTGCTGATCCTGC
	AGGCTGGGAAAAGTTAGGAGT
Ngf	CAGCATGGTCGAGTTTTG
	GATAGAAAGCTGCGTCCT
Bdnf	ACAAGACACATTACCTTCCAGC
	ACCTGGTGGAACATTGTGG
Bmp2	CGCAGACAACCCCAACATCA
	TGGAGTTCAGGTGGTCACAAGG
Runx2	CCACCAACTCCTTGCTGCTC
	GGAATAGCGTCAGGTAAGCGT
Ocn	CCAACTCTTTTGTGCCAGAGA
	GGCTACATTGGTGTTGAGCTTTT

Table S2. Vector information of Talin1 plasmid

Vector	information
vector name	GV657
element order	CMV enhancer-MCS-3flag-polyA-EF1A-zsGreen-sv40-puromycin
cloning site	BamHI / KpnI
