# Supplementary material for: Bidirectional integrin β1 activation synergizes neurovascular coupling and enhances bone regeneration
Source: Nat Commun. 2026 Jun 12;17:7610. doi: 10.1038/s41467-026-74071-3 (PMC13424096; doi:10.1038/s41467-026-74071-3)
Supplement: Supplementary file 2 — Reporting Summary [file 41467_2026_74071_MOESM2_ESM.pdf]

nature portfolio

Corresponding author(s): Jing Wang

Last updated by author(s): May 7, 2026

Reporting Summary

Nature Portfolio wishes to improve the reproducibility of the work that we publish. This form provides structure for consistency and transparency in reporting. For further information on Nature Portfolio policies, see our Editorial Policies and the Editorial Policy Checklist.

Statistics

For all statistical analyses, confirm that the following items are present in the figure legend, table legend, main text, or Methods section.

n/a	Confirmed	
☐	☒	The exact sample size (*n*) for each experimental group/condition, given as a discrete number and unit of measurement
☐	☒	A statement on whether measurements were taken from distinct samples or whether the same sample was measured repeatedly
☐	☒	The statistical test(s) used AND whether they are one- or two-sided<br>*Only common tests should be described solely by name; describe more complex techniques in the Methods section.*
☐	☒	A description of all covariates tested
☐	☒	A description of any assumptions or corrections, such as tests of normality and adjustment for multiple comparisons
☐	☒	A full description of the statistical parameters including central tendency (e.g. means) or other basic estimates (e.g. regression coefficient) AND variation (e.g. standard deviation) or associated estimates of uncertainty (e.g. confidence intervals)
☐	☒	For null hypothesis testing, the test statistic (e.g. *F*, *t*, *r*) with confidence intervals, effect sizes, degrees of freedom and *P* value noted<br>*Give P values as exact values whenever suitable.*
☒	☐	For Bayesian analysis, information on the choice of priors and Markov chain Monte Carlo settings
☒	☐	For hierarchical and complex designs, identification of the appropriate level for tests and full reporting of outcomes
☒	☐	Estimates of effect sizes (e.g. Cohen's *d*, Pearson's *r*), indicating how they were calculated

Our web collection on statistics for biologists contains articles on many of the points above.

Software and code

Policy information about availability of computer code

Data collection	Data collection described in Methods section. No custom software was used.
Data analysis	Data analysis is described in the Methods section. Statistical analysis was performed using SPSS software, version 23.0. Graphs were plotted using GraphPad Prism 9 version 9.5.1.

For manuscripts utilizing custom algorithms or software that are central to the research but not yet described in published literature, software must be made available to editors and reviewers. We strongly encourage code deposition in a community repository (e.g. GitHub). See the Nature Portfolio guidelines for submitting code & software for further information.

Data

Policy information about availability of data

All manuscripts must include a data availability statement. This statement should provide the following information, where applicable:

- Accession codes, unique identifiers, or web links for publicly available datasets
- A description of any restrictions on data availability
- For clinical datasets or third party data, please ensure that the statement adheres to our policy

Source data are provided with this paper. All data supporting the findings of this study are available within the article and its supplementary files.

Research involving human participants, their data, or biological material

Policy information about studies with human participants or human data. See also policy information about sex, gender (identity/presentation), and sexual orientation and race, ethnicity and racism.

Reporting on sex and gender	This work does not involve human research
Reporting on race, ethnicity, or other socially relevant groupings	This work does not involve human research
Population characteristics	This work does not involve human research
Recruitment	This work does not involve human research
Ethics oversight	This work does not involve human research

Note that full information on the approval of the study protocol must also be provided in the manuscript.

Field-specific reporting

Please select the one below that is the best fit for your research. If you are not sure, read the appropriate sections before making your selection.

☒ Life sciences ☐ Behavioural & social sciences ☐ Ecological, evolutionary & environmental sciences

For a reference copy of the document with all sections, see nature.com/documents/nr-reporting-summary-flat.pdf

Life sciences study design

All studies must disclose on these points even when the disclosure is negative.

Sample size	The animal sample size was estimated based on previous studies (doi:10.1021/acsnano.4c10430; 10.1016/j.bioactmat.2024.10.020). For all in vitro studies, at least three random samples were selected for statistical analysis in each experiment based on the effect size and overlap between distributions. For all in vivo studies, at least three random samples were applied for statistical analysis in each experiment.
Data exclusions	No data was excluded.
Replication	All experiments were performed at least in triplicate and all attempts at replication were successful.
Randomization	Different treatments were assigned to animals randomly divided into control and experimental groups. For histological, immunohistochemistry, and immunofluorescence staining, the field of view was randomly selected for analysis. For qRT-PCR, samples were randomized and analyzed with standard approaches. Cell culture samples were assigned randomly, with control and experimental groups analyzed in identical conditions to minimize potential covariates.
Blinding	For in vitro studies, blinding of test samples was not applicable since differences between groups were clear to the researchers by naked eye. However, the data were anonymized for statistical analysis. For in vivo studies, different researchers were randomly involved in animal group distribution, operation, and evaluation. All investigators were blinded to group allocation during data collection. All assessments of functional recovery were performed in a blinded manner. Immunostaining expression level was also performed blinded and the data were anonymized for statistical analysis

Reporting for specific materials, systems and methods

We require information from authors about some types of materials, experimental systems and methods used in many studies. Here, indicate whether each material, system or method listed is relevant to your study. If you are not sure if a list item applies to your research, read the appropriate section before selecting a response.

Materials & experimental systems

n/a	Involved in the study
☐	☒ Antibodies
☐	☒ Eukaryotic cell lines
☒	☐ Palaeontology and archaeology
☐	☒ Animals and other organisms
☒	☐ Clinical data
☒	☐ Dual use research of concern
☒	☐ Plants

Methods

n/a	Involved in the study
☒	☐ ChIP-seq
☒	☐ Flow cytometry
☒	☐ MRI-based neuroimaging

Antibodies

Antibodies used: NGF (Santa, sc-32300, 1:1000), BDNF (Santa, sc-65513, 1:1000), HIF-1α (Santa, sc-71247, 1:1000), VEGF (Santa, sc-7269, 1:1000), ITGB1 (abcam, ab183666, 1:1000), RUNX2 (Santa, sc-390351, 1:1000), BMP2 (Santa, sc-137087, 1:1000), OCN (Santa, sc-365797, 1:1000)

Validation: Validation statements in the manufacturer's website.

Eukaryotic cell lines

Policy information about cell lines and Sex and Gender in Research

Cell line source(s): human umbilical vein endothelial cell line (HUVEC) and the rat Schwann cell line (RSC96) were obtained from the Stem Cell Bank of the Chinese Academy of Sciences (Shanghai, China). The rat bone marrowmesenchymal stem cell (BMSC) were extracted from 3-week-old Sprague Dawley (SD) rat pups and cultured with α-MEM medium supplemented with 10% fetal bovine serum and 1% penicillin-streptomycin.

Authentication: HUVEC and RSC96 were authenticated by tem Cell Bank of the Chinese Academy of Sciences. IThe BMSCs were authenticated by testing differentiation to a osteoblast phenotype with osteogenic induction medium using the established protocol.

Mycoplasma contamination: We confirm that all cell lines were negative for mycoplasma contamination.

Commonly misidentified lines (See ICLAC register): There was no commonly misidentified lines for our study.

Animals and other research organisms

Policy information about studies involving animals; ARRIVE guidelines recommended for reporting animal research, and Sex and Gender in Research

Laboratory animals: 6-week-old SD male rats (200-250g) were used for in vivo evaluation of neurogenesis, angiogenesis, and osteogenesis.

Wild animals: The study did not involved wild animals

Reporting on sex: SD rats used in this study were male

Field-collected samples: The study did not involved samples collected from field.

Ethics oversight: All animal experiments were conducted in accordance with the guidelines of the Institutional Animal Care and Use Committee of The Third Affiliated Hospital of Air Force Medical University. The study protocol was approved under ethical approval number 2025 kq-006.

Note that full information on the approval of the study protocol must also be provided in the manuscript.

Plants

Seed stocks	NA
Novel plant genotypes	NA
Authentication	NA
