# Supplementary material for: Bidirectional integrin β1 activation synergizes neurovascular coupling and enhances bone regeneration
Source: Nat Commun. 2026 Jun 12;17:7610. doi: 10.1038/s41467-026-74071-3 (PMC13424096; doi:10.1038/s41467-026-74071-3)
Supplement: Supplementary file 3 — Transparent Peer Review file [file 41467_2026_74071_MOESM3_ESM.pdf]

nature portfolio

Peer Review File

Bidirectional integrin β1 activation synergizes neurovascular coupling and enhances bone regeneration

Corresponding Author: Professor Jing Wang

This file contains all reviewer reports in order by version, followed by all author rebuttals in order by version.

Attachments originally included by the reviewers as part of their assessment can be found at the end of this file.

Version 0:

Reviewer comments:

Reviewer #1

(Remarks to the Author)
NCOMMS-25-80494
Bidirectional integrin beta 1 activation synergizes neurovascular coupling and enhances bone regeneration.
Wu, An et al
The hypothesis is that concurrent activation of integrin beta 1 signaling can be used to promote neurovascular-bone network formation to accelerate repair pathways to repair critical sized bone defects, which currently remain difficult to repair due to limitation in biomaterial-,-bioactivating-pathways.

Fig 1. This is an enormous use of space that could be better used in the more complex images int he figures that follow. All figures are crowded, making it difficult to find panel designations. Consistent pattern in progression of images should be used: either horizontal or vertical progressions, but mixing these two should be avoided, as in Figure 4 and spelling error corrected, again as in fig 4 A.
Fig. 2. The bifunctional peptide for REDV-IKVAV uses a "GGG" linker. Presumably this is used to isolate and ensure correct folding for either of the two "outside in" signaling peptide pathways . Is there data, such as the use of either peptide alone to show a baseline and compare levels of the single peptide pathway compared to data for the bifunctional peptide?
The legend is not self-contained and rather requires the reader to return to the text to decipher. For example, in panels B and D, there are additional amino acid sequences provided for the bifunctional peptide that are not described. Is the reader encouraged to measure the extent of tube formation?
Panel H. How was HPLC used to provide a folding analysis of the peptide. The reader is provided to label to the content of the diagrams. Some panels, such as "I" is whimsical. Is there data for the efficiency of the plasmid
The authors must make the figure legends interpretable without reference back to the text of the manuscript. This is not yet the case for all 9-figure legend. There are numerous abbreviations or acronyms used that proved to be confusing. For the extensive work in this project, this is a defect.

The paracrine signaling is well documented and compelling evidence. The authors used siRNA to ablate the integrin beta signal axis. Such an approach to the conditioned media experiment should be considered. Is space is limited; the authors may consider using addendum material if the editor is agreeable.
The figures are very densely packed, and some panels are not necessary to tell the story. These unnecessary images may be eliminated to provide additional space for the remaining panels to better illustrate the manuscript, which deserves such consideration.

References could be extended beyond those cited, especially in areas that summarize the issues addressed. There sems to be a bias in authors cited.

Reviewer #2

(Remarks to the Author)
Recommendation: Reject (in current form)

This study presents an ambitious and timely approach in bone tissue engineering by proposing a scaffold that co-activates integrin 131 via both “outside-in” and “inside-out” signaling. The experimental work is extensive and covers multiple levels, from material characterization to in vivo models and single-cell sequencing, which is commendable. After careful evaluation , I find that the manuscript, in its current state, does not yet meet the high standard of novelty and scientific rigor required for publication in our journal. While the premise is promising, several critical concerns regarding experimental design, mechanistic justification, and data interpretation must be thoroughly addressed to substantiate the central claims.
Major Concerns:
The abstract lacks sufficient rigor:The abstract should more precisely reflect the conclusions that the current evidence can support. Until the key supplementary experiments are completed, it is not advisable to make overly strong mechanistic claims. For example, the focus could shift toward describing "the construction of a scaffold that simultaneously delivers peptides and plasmids, and the observation that it synergistically promotes nerve and vascular regeneration alongside bone repair." The notion of "achieving coupling through bidirectional ITGB1 activation" should be presented as a working hypothesis that remains to be verified.
Justification for Neural Marker Selection: The study utilizes NF200 and S100 as markers for nerve regeneration. However, a more nuanced discussion is needed. These are general structural markers and do not distinguish between sensory, sympathetic, or motor nerve subtypes. Given that current literature strongly emphasizes the specific and potent role of sensory nerves (e.g., via CGRP/SP signaling) in coupling with angiogenesis and osteogenesis, the reviewers question the rationale behind this choice. To strengthen the “neurovascular coupling” hypothesis, incorporating subtype-specific markers (e.g., CGRP for sensory nerves, TH for sympathetic nerves) in key experiments would be necessary to define the functional phenotype of the regenerated nerves.
Control for Endogenous Neural Interference: The impressive in vivo neuroregenerative effects attributed to the PTPG scaffold are compelling. However, the potential confounding influence of the host’s intrinsic nerve repair capacity needs to be addressed. To conclusively demonstrate that the scaffold is the primary driver, the inclusion of a more stringent control model, such as a chemical denervation model (e.g., using capsaicin to deplete sensory nerves or 6-OHDA for sympathetic nerves) prior to scaffold implantation, would significantly enhance the specificity and impact of the findings.
Rationale for Peptide Selection: The manuscript combines REDV/LXW7 (vascular) and IKVAV/YIGSR (neural) peptides. While these are known bioactive sequences, the scientific rationale for selecting this specific combination over other possible pairs, and the evidence that they indeed provide a synergistic effect on ITGB1 activation compared to single peptides, requires deeper justification. A more detailed discussion on the binding specificity to ITGB1 and the comparative advantages of this combination would clarify the design logic.
Clarity in Experimental Group Description: There is a notable omission in the Methods or Results section regarding the precise composition of the control groups (PG, PPG, TPG). A clear, explicit definition of what each acronym represents (e.g., PG = PLA-HA+GelMA; PPG = Peptide+PLA-HA+GelMA; TPG = Talin1 plasmid+PLA-HA+GelMA) is essential for reproducibility and for readers to accurately interpret the comparative data presented in figures like Figure 4.
Loading and characterization of peptides and plasmids: The method mentions "incorporating" the peptide and plasmid complexes into GelMA, but lacks details on the specific loading efficiency, encapsulation efficiency measurement methods, and data verifying bioactivity post-loading (e.g., does the secondary structure of the peptide change after loading? Is the integrity of the plasmid maintained?). The in vitro release curve alone is insufficient to prove their functional integrity. The core concept of this study is forward-thinking. However, critical issues in experimental design, control setup, and marker selection prevent the current data from fully substantiating the central claim of "neurovascular coupling." As significant gaps in scientific rigor and mechanistic depth remain, I recommend rejecting the manuscript in its present form. Should the authors address the outlined concerns through substantial revisions and additional experiments in the future, this promising line of research could yield more robust and impactful findings.

Reviewer #3

(Remarks to the Author)
Wu et al. reported their work on the development of a multifunctional scaffold, PTPG, for bone regeneration here. The research is of great significance, given that the regeneration of bone with large defects is a long-standing problem. Their design of PTPG is interesting as it focuses on activating integrin 131 bidirectionally. While ITGB1 has been investigated for promoting bone regeneration, constructing a scaffold that can regulate ITGB1 both from the outside and within cells is considered a new approach and may potentially address this medical challenge. The experiment design is thorough and logical, encompassing material development, comprehensive in vitro and in vivo assays, and single-cell profiling. The results presented here are promising and supportive of the overall conclusion. I recommend publication in the prestigious journal Nature Communications, with the following revisions.

1. The language needs improvement, and abbreviations in the abstract should be defined or spelled out.
2. Line 108, there is no Fig. S1G.
3. Fig 1. What is the highly branched Poly? While it is mentioned in the main text as “Highly branched poly(13-amino esters),” it should be labeled in the figure and elaborated upon in the figure caption.
4. In Figure 2K and M, it should be poly(13-amino esters), not 13-amino esters, as they are polymers, not monomers. What is “con”?
5. Lines 109-111, a statistical analysis between the four groups is needed to support that statement.
6. Line 113: The molecular docking modeling methods should be described in the Methods section.
7. Can the authors confirm the scale bars of Figures 3D, E, and F?
8. Line 171, What is CLSM? Fig 5B-G, what is NC?
9. Line 192, The method for siRNA-mediated knockdown of ITGB1 should be provided.

10. Line 370, The sources and purity of the PLA and HA should be given in the materials section.
11. Lin 378, In the methods, the hyperbranched polymer is too broad. The name and basic structural parameters (e.g., molecular weights) of the polymers used should be given.
12. Line 630, Fig 2A. The chemical structure of the final conjugated peptides should be given. There are multiple amines and carbolic acid groups. It is unclear how the conjugations are done and what the structure of the final products is.
13. In the Supporting Information, in Fig. S1, the theoretical molar mass should also be given. Also, the different ions shown in the mass should be analyzed.
14. In the Supporting Information, in Fig. S1, the authors should specify the type of mass spectrometry and the gradient of the HPLC method in the methods section.
15. Line 382, What is the intensity of UV used in fabricating the PLA-HA scaffolds?
16. The approval of the animal protocol by the Institutional Animal Care and Use Committee should be provided.
17. Fig. 7 and 8, 4 weeks and 8 weeks should be labeled as the euthanization time points, not the intervals between events.
18. A critical discussion of the limitations of the study is recommended.

Version 1:

Reviewer comments:

Reviewer #2

(Remarks to the Author)
I thank the authors for their thorough and timely revision of the manuscript. They have carefully addressed all my previous concerns, with detailed responses provided in the "Response to Reviewers" letter. The manuscript is now suitable for publication in Nature Communications.

The only remaining requirement is that the authors supply unmodified, fully uncropped original raw files for all Western blot data presented in the article.

Upon the author making minor revisions to address the aforementioned improvement, the manuscript could be accepted for publication.

Reviewer #3

(Remarks to the Author)
Most of my concerns have been addressed now.

One minor note is the Fig. 2A, the use of arrows between two reactive groups is confusing. The box on the IKVAV should be on an amine group, not on the methyl group. It is difficult to control which amines or carboxylic acids (side or terminal) to react. I recommend changing the related phases in the legend.

Open Access This Peer Review File is licensed under a Creative Commons Attribution 4.0 International License, which

permits use, sharing, adaptation, distribution and reproduction in any medium or format, as long as you give appropriate credit to the original author(s) and the source, provide a link to the Creative Commons license, and indicate if changes were made.
In cases where reviewers are anonymous, credit should be given to 'Anonymous Referee' and the source.
The images or other third party material in this Peer Review File are included in the article’s Creative Commons license, unless indicated otherwise in a credit line to the material. If material is not included in the article’s Creative Commons license and your intended use is not permitted by statutory regulation or exceeds the permitted use, you will need to obtain permission directly from the copyright holder.
To view a copy of this license, visit https://creativecommons.org/licenses/by/4.0/

Bidirectional integrin β1 activation synergizes neurovascular coupling and enhances bone regeneration (NCOMMS-25-80494)

Responses to reviewers' comments

Reviewer #1:

Comment 1: Fig 1. This is an enormous use of space that could be better used in the more complex images in the figures that follow. All figures are crowded, making it difficult to find panel designations. Consistent pattern in progression of images should be used: either horizontal or vertical progressions, but mixing these two should be avoided, as in Figure 4 and spelling error corrected, again as in fig 4 A.

Response: We appreciate the reviewer's comments on space efficiency and figure presentation, which guided us to improve the clarity and presentation of the figures. In response, all figures were revised with the following changes:

1. Panels that did not contribute essential information to the main conclusions were relocated to the Supplementary Information.
2. Spacing between adjacent panels was increased to reduce crowding and improve separation of panel boundaries and designations.
3. Panel progression was standardized to a single left-to-right horizontal sequence across all multi-panel figures to avoid mixed horizontal and vertical layouts (including Figure 4).

In addition, all figures and figure legends were re-checked to ensure consistent panel labeling and to remove any remaining spelling or grammatical errors.

Comment 2: Fig. 2. The bifunctional peptide for REDV-IKVAV uses a "GGG" linker. Presumably this is used to isolate and ensure correct folding for either of the two "outside in" signaling peptide pathways. Is there data, such as the use of either peptide alone to show a baseline and compare levels of the single peptide pathway compared to data for the bifunctional peptide?

Response: Thank you for this comment. We recognize the importance of verifying that each individual peptide retains activity after modification and that covalent linkage does not diminish function, as well as to determine whether the bifunctional construct provides additive or synergistic effects. The use of a short glycine linker (GGG) is intended to provide flexible spacing with

minimal steric hindrance, helping to reduce mutual interference between adjacent functional motifs and supporting independent receptor engagement. Consistent with this rationale, tri-glycine linkers have been used in peptide and conjugate design to preserve binding or bioactivity and to enable multi-functional behavior.[1–3]

To address the reviewer's request for baseline comparisons, the activity of REDV alone and IKVAV alone was evaluated alongside the covalently linked REDV-IKVAV peptide. Relative to the single-peptide controls, REDV-IKVAV significantly enhanced Schwann cell and endothelial cell proliferation and migration, and significantly increased ITGB1 expression. These findings confirmed that the covalently linked bifunctional peptide remains functional and produces the intended biological effects. The corresponding control and comparison data are provided in **Figure S3** (Supplementary Information), and the related description has been added to the main text (**Lines 109–110 and 128–132**).

[1] Wu D, Tian W, Li J, Zhang Q, Wang H, Zhang L, Xie Z, Ji A, Li Y. Peptide P11 suppresses the growth of human thyroid carcinoma by inhibiting the PI3K/AKT/mTOR signaling pathway. Mol Biol Rep. 2019 Jun;46(3):2665-2678. doi: 10.1007/s11033-019-04698-7.

[2] More SH, Schmutz M, Jierry L, Ganesh KN. Supramolecular multiplexes from collagen mimetic peptide-PNA(GGG) conjugates and C-rich DNA: pH-induced reversible switching from triplex-duplex to triplex-*i*-motif. Biomater Sci. 2024 Dec 17;13(1):261-274. doi: 10.1039/d4bm00955j.

[3] Šulc J, Vondrášek J. Unraveling the Tether: Exploring Representative Protein Linkers and Their Structural and Thermodynamical Properties. J Phys Chem B. 2025 Apr 17;129(15):3720-3730. doi: 10.1021/acs.jpcb.4c04194.

Comment 3: The legend is not self-contained and rather requires the reader to return to the text to decipher. For example, in panels B and D, there are additional amino acid sequences provided for the bifunctional peptide that are not described. Is the reader encouraged to measure the extent of tube formation?

<u>Response</u>: We agree that figure legends should be fully self-contained. All relevant figure legends have been comprehensively revised to improve clarity and completeness.

Specifically, the full amino acid sequences of the bifunctional and related peptides have now been explicitly included in the legend to Panels B and D. The sequences are provided as follows: LXW7-YIGSR (Cyclo-Arg-Gly-Asp-Gly-Gly-Gly-Tyr-Ile-Gly-Ser-Arg), LXW7-IKVAV (Cyclo-Arg-Gly-Asp-Gly-Gly-Gly-Ile-Lys-Val-Ala-Val), REDV-IKVAV (Arg-Glu-Asp-Val-Gly-Gly-Gly-Ile-Lys-Val-Ala-Val), and REDV-YIGSR (Arg-Glu-Asp-Val-Gly-Gly-Gly-Tyr-Ile-Gly-Ser-Arg) **(Lines 696–698).** This revision ensures that the biochemical composition and structural design of each peptide construct are transparent and interpretable directly from the legend.

Regarding tube formation, quantitative analyses were indeed performed. The extent of angiogenic tube formation was evaluated using standard metrics, including total tube length and number of branch points. These quantitative results are presented in **Figure S2D** (Supplementary Information), and this information has now been clearly stated in the revised legend to avoid ambiguity.

Comment 4: Panel H. How was HPLC used to provide a folding analysis of the peptide. The reader is provided to label to the content of the diagrams. Some panels, such as "I" is whimsical. Is there data for the efficiency of the plasmid

<u>Response</u>: (1) Role of HPLC and folding analysis

We apologize for the lack of clarity in the original legend. Reverse-phase high-performance liquid chromatography (RP-HPLC) was not used to directly examine peptide folding. Rather, it was employed to evaluate purity and conformational homogeneity of the synthesized bifunctional peptide. In the present study, the relative purity of REDV-IKVAV reached 95%, meeting the requirements for subsequent biological experiments. The presence of a single sharp peak in the chromatogram indicates a homogeneous peptide population, supporting high purity and consistent molecular composition. TheRP-HPLC results are presented in **Figure S1**.

Circular dichroism (CD) spectroscopy was used to examine secondary structure and folding state. The CD spectrum showed a strong positive signal near 190 nm, a distinct negative trough in the 200 to 230 nm range, and minor fluctuations in the 240 to 260 nm region. These features indicate a conformationally ordered structure with stable secondary structural elements. Importantly, no fundamental change in secondary structure was observed before and after release. The CD results are presented in **Figure S4E**, and the corresponding clarification has been added to the revised text **(Lines 114 to 116 and 170 to 182)**.

(2) Panel I presentation

This panel was originally designed as a schematic illustration of the hypothesized talin1-mediated inside-out signaling mechanism. We agree that the simplified cartoon style may have appeared overly conceptual. In response, the panel has been redesigned with clearer annotations and direct labeling of the molecular events represented. The revised panel ensures that each graphical element corresponds to experimentally supported findings described in the manuscript.

(3) Plasmid efficiency

Direct quantitative transfection efficiency was not measured using flow cytometry or reporter assays. However, functional validation of plasmid delivery and biological activity was rigorously assessed through complementary approaches:

1. Immunofluorescence staining at 48 hours **(Figure 2G and 2I)** demonstrated robust and specific Talin1 expression in transfected cells.
2. Quantitative RT-PCR confirmed statistically significant upregulation of Talin1 mRNA in the transfected group **(Figure 2H and 2J).**
3. Agarose gel electrophoresis showed no detectable difference in band size between the released plasmid and the original plasmid, confirming structural integrity after release **(Figure S4F)**.

These results collectively provided consistent evidence that the plasmid was successfully delivered and biologically active in the present experimental system.

Comment 5: The authors must make the figure legends interpretable without reference back to the text of the manuscript. This is not yet the case for all 9-figure legend. There are numerous abbreviations or acronyms used that proved to be confusing. For the extensive work in this project, this is a defect.

<u>Response</u>: All nine figure legends have been comprehensively revised to improve clarity, completeness, and readability. Specifically:

1. All abbreviations and acronyms appearing in the figures are now defined in full at their first occurrence within each legend, even if previously defined in the main text.
2. Experimental conditions, sample groups, and treatment parameters are now explicitly described in the legends.
3. Quantification methods, statistical analyses, and significance indicators are clearly explained.

4. Panel descriptions have been expanded to allow readers to understand the data and conclusions directly from the legend.

We have carefully reviewed each legend to ensure consistency in terminology and to eliminate ambiguity. We believe these revisions substantially improve the interpretability and presentation quality of the figures.

Comment 6: The paracrine signaling is well documented and compelling evidence. The authors used siRNA to ablate the integrin beta signal axis. Such an approach to the conditioned media experiment should be considered. Is space is limited; the authors may consider using addendum material if the editor is agreeable.

Response: We agree that paracrine signaling is a critical component of the biological mechanism proposed in this study, and that targeted interruption of the integrin β signaling axis in the conditioned media model would further strengthen mechanistic interpretation.

In the present manuscript, siRNA-mediated knockdown of integrin β was performed to validate its role in direct signaling responses. However, we acknowledge that extending this approach to the conditioned media experiments would provide additional mechanistic clarity regarding the contribution of the integrin β axis to paracrine communication.

It is necessary to point out that the paracrine crosstalk between neural cells, vascular endothelial cells, and bone marrow mesenchymal stem cells (BMSCs) cultured on the composite scaffold remains a central and novel aspect of this work. The conditioned media experiments are critical for demonstrating that the scaffold promotes osteogenic differentiation of BMSCs through neurovascular-mediated signaling pathways. For this reason, we consider these data essential to the mechanistic narrative and therefore appropriate for inclusion in the main text rather than relegation to supplementary material.

If the editor considers space limitations a concern, we would be willing to move certain supporting mechanistic validation data, including extended knockdown analyses, to supplementary material to maintain balance between completeness and readability.

Comment 7: The figures are very densely packed, and some panels are not necessary to tell the story. These unnecessary images may be eliminated to provide additional space for the remaining panels to better illustrate the manuscript, which deserves such consideration.

<u>Response</u>: Thank you for this constructive suggestion. We have removed non-essential panels across the figures and adjusted the layout to provide more space for the remaining images, improving readability and presentation.

Comment 8: References could be extended beyond those cited, especially in areas that summarize the issues addressed. There sems to be a bias in authors cited.

<u>Response</u>: We have carefully expanded and diversified the reference list, particularly in sections that summarize key concepts and contextual issues. Additional foundational and recent studies from a broader range of research groups have been incorporated to improve balance and reduce any potential author bias.

Reviewer #2:

Comment 1: The abstract lacks sufficient rigor: The abstract should more precisely reflect the conclusions that the current evidence can support. Until the key supplementary experiments are completed, it is not advisable to make overly strong mechanistic claims. For example, the focus could shift toward describing "the construction of a scaffold that simultaneously delivers peptides and plasmids, and the observation that it synergistically promotes nerve and vascular regeneration alongside bone repair." The notion of "achieving coupling through bidirectional ITGB1 activation" should be presented as a working hypothesis that remains to be verified.

<u>Response</u>: We have revised the abstract to remove overly strong mechanistic statements and to ensure it reflects only what the current data directly support. The scaffold's co-delivery of peptides and plasmid and the observed enhancement of neurovascular regeneration and bone repair are now presented as the primary findings. Bidirectional ITGB1 activation is framed as a working hypothesis requiring further validation. These changes are incorporated in the revised abstract **(Lines 27–30 and 39–41)**.

Comment 2: Justification for Neural Marker Selection: The study utilizes NF200 and S100 as markers for nerve regeneration. However, a more nuanced discussion is needed. These are general

structural markers and do not distinguish between sensory, sympathetic, or motor nerve subtypes. Given that current literature strongly emphasizes the specific and potent role of sensory nerves (e.g., via CGRP/SP signaling) in coupling with angiogenesis and osteogenesis, the reviewers question the rationale behind this choice. To strengthen the "neurovascular coupling" hypothesis, incorporating subtype-specific markers (e.g., CGRP for sensory nerves, TH for sympathetic nerves) in key experiments would be necessary to define the functional phenotype of the regenerated nerves.

<u>Response</u>: In the original design, NF200 and S100 were selected as first-line markers to establish whether neural ingrowth and peripheral nerve associated cellular components were present within the defect region after scaffold implantation. This approach provided an initial and robust assessment of overall nerve presence and structural regeneration. However, we acknowledge that this level of analysis is not sufficient to determine whether the regenerated neural network includes sensory and sympathetic components that are most relevant to functional neurovascular coupling. To address this concern directly, we have now incorporated subtype-specific markers in the *in vivo* experiments. Specifically, calcitonin gene-related peptide (CGRP) was used as a marker for sensory nerves, and tyrosine hydroxylase (TH) was used as a marker for sympathetic nerves. In the revised experiments, the PTPG scaffold group exhibited significantly increased CGRP-positive and TH positive signals at the implant site compared with the other groups. These findings indicate that the scaffold promotes regeneration of functional nerve subtypes, rather than only reestablishing non-specific neural structures.

Consistent with current understanding, the increased CGRP positive sensory innervation is likely to contribute to neurovascular mediated osteogenesis through neuropeptide signaling pathways (including CGRP and substance P related signaling), and the regenerated TH positive sympathetic fibers may participate in regulating local vascular tone, inflammatory status, and tissue microenvironment homeostasis. We have revised the manuscript to present these mechanistic links with appropriate caution, describing them as biologically plausible interpretations supported by prior literature rather than as definitive causal proof from the present dataset.

The new results and discussion have been added to the main text **(Lines 298 to 302)**. The corresponding immunofluorescence images and quantitative statistical analyses are provided in the Supplementary Information **(Figures S9 and S10)**.

Comment 3: Control for Endogenous Neural Interference: The impressive in vivo neuroregenerative effects attributed to the PTPG scaffold are compelling. However, the potential confounding influence of the host's intrinsic nerve repair capacity needs to be addressed. To conclusively demonstrate that the scaffold is the primary driver, the inclusion of a more stringent control model, such as a chemical denervation model (e.g., using capsaicin to deplete sensory nerves or 6-OHDA for sympathetic nerves) prior to scaffold implantation, would significantly enhance the specificity and impact of the findings.

Response: We agree that distinguishing scaffold-driven neuroregeneration from the host's intrinsic repair capacity is essential to strengthen mechanistic specificity. Given that the current literature has clearly emphasized the specific and powerful role of sensory nerves in angiogenesis and osteogenesis, we supplemented our study with a chemical denervation model using capsaicin pretreatment to deplete sensory nerves. This serves as a more rigorous control for evaluating the neurovascular regenerative effects of the PTPG scaffold. Male SD rats (2 months old) were used. Under sterile conditions, the dorsal musculature was dissected and the ligaments of the vertebral articular surfaces were transected to expose the L3–L5 dorsal root ganglia (DRG). Capsaicin was locally administered to the DRG for three consecutive days with incremental dosing (30 mg/kg on Day 1, 50 mg/kg on Day 2, and 70 mg/kg on Day 3) to achieve selective sensory nerve depletion. Scaffolds were implanted after confirmation of the denervation model. The experimental groups were defined as: blank group, PTPG group, capsaicin + blank group, and capsaicin + PTPG group. The results demonstrated that, compared with the PTPG group, the capsaicin + PTPG group exhibited a significant reduction in CGRP-positive sensory fibers and EMCN-positive vascular structures. Importantly, no significant difference was observed between the capsaicin + PTPG group and the blank group, and the capsaicin + blank group showed the lowest levels of CGRP and EMCN among all groups. These findings indicate that depletion of sensory nerves markedly attenuated the scaffold-induced neurovascular regeneration.

Taken together, these data demonstrate that the enhanced neuroangiogenic effects observed in the PTPG group are not solely attributed to endogenous repair mechanisms. Rather, the effects depend on functional sensory nerve regeneration promoted by the scaffold. The comparable expression levels in the Capsaicin and Blank groups support the specificity of the denervation model and suggest that capsaicin treatment did not introduce confounding non-specific tissue effects.

These additional results have been incorporated into the revised manuscript **(Lines 312–319),** the detailed protocol has been added to the Supplementary Methods **(Lines 304–312),** and the corresponding immunofluorescence images, quantitative analyses, and statistical data are presented in **Figure S11.**

Comment 4: Rationale for Peptide Selection: The manuscript combines REDV/LXW7 (vascular) and IKVAV/YIGSR (neural) peptides. While these are known bioactive sequences, the scientific rationale for selecting this specific combination over other possible pairs, and the evidence that they indeed provide a synergistic effect on ITGB1 activation compared to single peptides, requires deeper justification. A more detailed discussion on the binding specificity to ITGB1 and the comparative advantages of this combination would clarify the design logic.

<u>Response</u>: Selection of the REDV–IKVAV combination was based on both biochemical considerations and functional screening results.

First, from a physicochemical standpoint, the candidate peptides (REDV, LXW7, IKVAV, and YIGSR) did not show substantial differences in overall hydrophilicity or charge distribution under physiological conditions, minimizing confounding effects related to surface adsorption or electrostatic bias. Therefore, the final selection was not dictated by gross chemical disparities, but rather by biological performance.

Second, functional assays were conducted to compare the biological effects of the candidate peptides on endothelial cells (ECs) and Schwann cells (SCs). Among the tested combinations, REDV–IKVAV demonstrated the most pronounced enhancement in EC and SC proliferation and migration. These results suggested complementary bioactivity toward vascular and neural cell populations.

To further substantiate the integrin-related mechanism, ITGB1 protein expression was examined by western blot. Compared with the other peptide combinations, REDV–IKVAV induced significantly higher ITGB1 expression. Importantly, comparative analyses between the individual peptides and the covalently linked conjugate demonstrated that REDV–IKVAV more effectively upregulated ITGB1 than REDV or IKVAV alone. This supports the presence of a functional enhancement beyond single-peptide activity.

Mechanistically, REDV is known to preferentially interact with endothelial-associated integrins, and IKVAV interacts with neural-related integrin receptors. The convergence of these motifs

within a single construct increases the probability of engaging ITGB1-associated heterodimers across both vascular and neural cell types. While we do not claim definitive receptor-binding specificity at the atomic level, our data support that this combination produces stronger ITGB1 activation at the cellular level compared with single motifs.

Based on the dual validation of cellular functional assays and ITGB1 expression analysis, REDV–IKVAV was selected for scaffold functionalization and subsequent *in vitro* and *in vivo* experiments. The relevant comparative data and discussion have been incorporated into the revised manuscript **(Lines 123–124 and 128–132).** The corresponding control and comparison data are provided in **Figure S3** (Supplementary Information).

Comment 5: Clarity in Experimental Group Description: There is a notable omission in the Methods or Results section regarding the precise composition of the control groups (PG, PPG, TPG). A clear, explicit definition of what each acronym represents (e.g., PG = PLA-HA+GelMA; PPG = Peptide+PLA-HA+GelMA; TPG = Talin1 plasmid+PLA-HA+GelMA) is essential for reproducibility and for readers to accurately interpret the comparative data presented in figures like Figure 4.

Response: We have revised the manuscript to clearly define all experimental groups in the Methods section and to ensure consistent usage throughout the Results and figure legends. The groups are now explicitly stated as follows:

PG = PLA-HA/GelMA

PPG = Peptide/PLA-HA/GelMA

TPG = Talin1 plasmid/PLA-HA/GelMA

PTPG = Peptide/ Talin1 plasmid/PLA-HA/GelMA

In addition, all abbreviations are now fully defined at first mention within the corresponding figure legends to avoid any ambiguity during figure-only reading. These revisions have been incorporated into the revised manuscript **(Lines 431–433 and 732–733).**

Comment 6: Loading and characterization of peptides and plasmids: The method mentions "incorporating" the peptide and plasmid complexes into GelMA, but lacks details on the specific loading efficiency, encapsulation efficiency measurement methods, and data verifying bioactivity post-loading (e.g., does the secondary structure of the peptide change after loading? Is

the integrity of the plasmid maintained?). The in vitro release curve alone is insufficient to prove their functional integrity.

Response: We have supplemented detailed experimental methods and corresponding data addressing loading efficacy, peptide structural stability, and plasmid integrity and activity after encapsulation and release.

1. Loading and encapsulation efficacy

Peptide and plasmid loading within GelMA hydrogels were evaluated using laser scanning confocal microscopy (LSCM). Peptides were fluorescently labeled (red) and plasmids were labeled (green) prior to mixing with the GelMA precursor solution. After photo-crosslinking, hydrogels were imaged at multiple randomly selected regions. The uniform spatial distribution of both fluorescent signals throughout the hydrogel matrix indicates efficient encapsulation and homogeneous incorporation **(Figure 3F; Lines 169–172).**

We have additionally quantified encapsulation efficacy by measuring the initial input amount and the released total amount after crosslinking. The release experiment was conducted for 40 days and terminated when no peptide or Talin 1 was detected in the supernatant. The final encapsulation efficiency was calculated as the ratio of the total released amount to the initially loaded amount. The loading efficiency exceeded 80% for peptide and Talin1 plasmids, demonstrating effective incorporation into the hydrogel network **(Figure S4D; Lines 176).**

2. Peptide secondary structure and post-release bioactivity

To determine whether hydrogel encapsulation altered peptide conformation, circular dichroism (CD) spectroscopy was performed on both the original peptide and the peptide recovered from *in vitro* release supernatants. Spectra were recorded in the 190–260 nm range, and secondary structure composition was analyzed using CDPro software.

The released peptide exhibited a CD spectral profile highly consistent with that of the original peptide, with no significant change in the relative proportions of α-helix, β-sheet, or random coil structures **(Figure S4E; Lines 179–182).** These findings indicate that encapsulation and release did not disrupt secondary structure.

Furthermore, functional assays performed with released peptide confirmed preserved biological activity, as evidenced by sustained promotion of endothelial and Schwann cell proliferation and migration relative to controls. These results collectively support retention of structural and functional integrity **(Figure 3)**.

3. Plasmid integrity and biological activity

Plasmid integrity following release was assessed using agarose gel electrophoresis. Plasmids collected from release supernatants displayed a single sharp band with electrophoretic mobility identical to that of the original plasmid **(Figure S4F; Lines 182–184).** This finding is indicative of structural integrity without detectable degradation.

Biological experiments demonstrated that the scaffold loaded with the talin1 plasmid exhibited upregulated expression of angiogenesis- and neurogenesis-related genes compared with the control group, confirming that the released plasmid retained its biological activity **((Figure 3).**

These additional structural, biochemical, and functional data provide a comprehensive validation that peptide and plasmid loading into GelMA preserves molecular integrity and bioactivity beyond simple release profiling.

Reviewer #3:

Comment 1: The language needs improvement, and abbreviations in the abstract should be defined or spelled out.

<u>Response</u>: We have carefully revised the manuscript to improve overall language clarity and fluency. The text was professionally edited by a native English-speaking expert with expertise in biomaterials research to ensure precision and readability.

In addition, all abbreviations appearing in the abstract are now defined at first mention. For example, REDV–IKVAV is now presented with its full amino acid sequence (Arg–Glu–Asp–Val– Gly–Gly–Gly–Ile–Lys–Val–Ala–Val) at first occurrence **(Line 31)**. We have also reviewed the abstract to ensure that other technical abbreviations are fully spelled out before being abbreviated.

Comment 2: Line 108, there is no Fig. S1G.

<u>Response</u>: The citation error in Line 108 (“Fig. S1G”) has been corrected to “Fig. S1”.

Comment 3. Fig 1. What is the highly branched Poly? While it is mentioned in the main text as "Highly branched poly(13-amino esters)," it should be labeled in the figure and elaborated upon in the figure caption.

Response: In the revised Figure 1, we have explicitly labeled the highly branched polymer as hyperbranched poly (13-amino ester)s (HPAEs). Furthermore, we also carefully examined the mention of the text, images and captions, and made necessary revisions **(Line 93, Line 672).**

Comment 4: In Figure 2K and M, it should be poly (13-amino esters), not 13-amino esters, as they are polymers, not monomers. What is "con"?

Response: Thank you for pointing out this error. In Figure 2, we have renamed the "13-amino ester" group as the "Talin1 plasmid" group. According to your suggestions, we have also clarified the abbreviation "con" in the figure legend. con = control, it represents the group without talin1 plasmid. The revised figures and corresponding legends are updated in **Line 672** of the revised manuscript.

Comment 5: Lines 109-111, a statistical analysis between the four groups is needed to support that statement.

Response: We have now added a formal statistical comparison across all four peptide-treated groups. Specifically, cell proliferation data were analyzed using one-way analysis of variance (one-way ANOVA), followed by Tukey's post-hoc multiple-comparisons test.

The results were as follows:

1. Within-group comparisons versus the 0 μM control: Both REDV–YIGSR and REDV–IKVAV significantly increased proliferation of HUVECs and RSC96 cells compared with the 0 μM group ($p < 0.05$).

0. Between-group comparisons among the four peptide-treated groups: No statistically significant differences in proliferation were detected among the four peptide groups for either HUVECs or RSC96 cells ($p > 0.05$).

These statistical results have been incorporated into the revised manuscript to substantiate the statement in **Lines 117–120.**

Comment 6: Line 113: The molecular docking modeling methods should be described in the Methods section.

Response: We have now added a detailed description of the molecular docking workflow to the Methods section to ensure transparency and reproducibility.

Briefly, the predicted structure of integrin 131 was obtained using AlphaFold. Ligands were prepared by assigning protonation states at pH 7.4 and generating 3D conformations using Open Babel. Receptor and ligand preparation and parameterization were performed with AutoDockTools (ADT3). Docking grid files were generated using AutoGrid, and docking simulations were carried out with AutoDock Vina (v1.2.0). The optimal binding pose was selected based on docking score and interaction geometry and was then used for interaction analysis. Visualization was performed in PyMOL, with integrin 131 displayed as a cartoon model and ligands shown as stick representations. Hydrogen bonds, ionic interactions, and hydrophobic interactions were displayed using dashed lines."

Full methodological details have been added to the Supplementary Methods **(Lines 178–188).**

Comment 7: Can the authors confirm the scale bars of Figures 3D, E, and F?

Response: The updated figures have been provided in the revised manuscript. Figure 3D (scale bar = 10 μm); Figure 3E (scale bar = 2 μm). Image 3F has been moved to Supplementary Figure S4C (scale bar = 10 μm).

Comment 8: Line 171, What is CLSM? Fig 5B-G, what is NC?

Response: In line 171, CLSM refers to confocal laser scanning microscopy. This term is now spelled out at first mention in the main text. In Figures 5B–G, NC refers to negative control, defined as the group without itgb1 siRNA treatment. This definition has now been added directly to the corresponding figure legends.

Comment 9: Line 192, The method for siRNA-mediated knockdown of ITGB1 should be provided.

Response: We have now added the full siRNA knockdown protocol for ITGB1 to the Materials and methods to ensure reproducibility.

Briefly, the ITGB1 siRNA and non-targeting negative control (NC) were designed and synthesized by Gene create Co., Ltd. (Wuhan, China). Transfections were carried out using Lipofectamine 3000 (Invitrogen, USA) following the manufacturer's instructions with minor modifications. Specifically, 5 μL of 20 μM ITGB1 siRNA (or NC) was diluted in 125 μL Opti-MEM Reduced Serum Medium and mixed with an equal volume of Lipofectamine 3000 diluted in Opti-MEM after 5 minutes. The siRNA-lipid complexes were incubated at room temperature for 15 minutes and then added dropwise to cells in 6-well plates at 60–70% confluency in antibiotic-free complete medium. Cells were incubated at 37°C in 5% CO2 for 6 hours, after which the medium was replaced with fresh complete medium supplemented with 10% fetal bovine serum and 1% penicillin-streptomycin.

The complete protocol has been added to the Supplementary Methods **(Lines 251–261).**

Comment 10: Line 370, The sources and purity of the PLA and HA should be given in the materials section.

<u>Response</u>: We have now specified the sources and relevant material specifications in the Materials section to improve transparency and reproducibility. Polylactic acid (PLA; Aladdin) with an average molecular weight of approximately 60,000, and hyaluronic acid (HA; Aladdin) with a stated purity of at least 97%, have been added to the manuscript **(Lines 407 and 411).**

Comment 11: Lin 378, In the methods, the hyperbranched polymer is too broad. The name and basic structural parameters (e.g., molecular weights) of the polymers used should be given.

<u>Response</u>: The Materials and methods section has been revised to specify the polymer identity and key structural parameter. The hyperbranched polymer used is hyperbranched poly (β-amino ester)s (HPAEs), with a molecular weight of approximately 14.8 kDa (as reported by the supplier/manufacturer). This information has now been added to the revised manuscript.

Comment 12: Line 630, Fig 2A. The chemical structure of the final conjugated peptides should be given. There are multiple amines and carbolic acid groups. It is unclear how the conjugations are done and what the structure of the final products is.

<u>Response</u>: In this study, the REDV–GGG–IKVAV peptide was conjugated to the polymer backbone through a carbodiimide-mediated amide coupling reaction. Specifically, the reaction was

designed to form an amide bond between the N-terminal amine of the peptide and activated carboxyl groups on the polymer.

With respect to functional groups on the peptide, REDV–GGG–IKVAV contains multiple amine functionalities (including the N-terminus and lysine side-chain amines) and at least one carboxyl functionality (C-terminus and/or acidic side chains). In our coupling strategy, only the N-terminal amine was used as the intended nucleophile to react with polymer carboxyl groups. The remaining side-chain amines and carboxyl groups were not targeted for coupling and therefore remain unreacted in the final conjugate.

To eliminate ambiguity for the reader, we have revised Figure 2A and the corresponding legend to include a clear chemical schematic of the final conjugated structure. The schematic explicitly indicates: (i) the polymer backbone bearing carboxyl groups, (ii) carbodiimide activation, (iii) the site-specific formation of an amide linkage at the peptide N-terminus, and (iv) the functional groups that remain free after conjugation. We have also clarified this coupling logic in the Methods section to ensure reproducibility.

Comment 13: In the Supporting Information, in Fig. S1, the theoretical molar mass should also be given. Also, the different ions shown in the mass should be analyzed.

Response: We have revised Figure S1 and the accompanying text in the Supporting Information to include the theoretical molar mass for each peptide and to provide interpretation of the major ion species observed in the mass spectra.

The theoretical molecular weights are now provided as follows: LXW7–YIGSR, 1568.69; LXW7–IKVAV, 1502.71; REDV–IKVAV, 1199.65; and REDV–YIGSR, 1265.33 **(Lines 112–114).**

In addition, the ion assignments have been clarified. Using REDV–IKVAV as an example, the main peak at m/z 1199.65 corresponds to the singly charged [M+H]+ ion and matches the theoretical mass. The peak at m/z 599.83 corresponds to the doubly charged $[M+2H]^{2}+$ species, and the peak at m/z 1281.65 is assigned to the sodium adduct [M+Na]+. These peaks collectively support successful synthesis and confirm the expected molecular composition of the peptide **(Lines 112–114).**

Comment 14: In the Supporting Information, in Fig. S1, the authors should specify the type of mass spectrometry and the gradient of the HPLC method in the methods section.

Response: We have updated the Supporting Information Methods to explicitly specify both the mass spectrometry modality and the RP-HPLC gradient conditions used for Figure S1.

For RP-HPLC, separation was performed using a COSMOSIL packed column (4.6 × 250 mm, 5 μm). The mobile phases were Solvent A (0.1% trifluoroacetic acid in acetonitrile) and Solvent B (0.1% trifluoroacetic acid in water). A linear gradient was applied as follows: 13% A/87% B at 0.0 min, 38% A/62% B at 25.0 min, 100% A/0% B at 25.1 min, and the run was stopped at 30.0 min. The flow rate was 1.0 mL/min, injection volume was 20 μL, and UV detection was performed at 220 nm (Supplementary Methods **Lines 189–196**).

For mass spectrometry, analysis was conducted using positive-mode electrospray ionization (ESI) with an m/z scan range of 300–2000. Full-scan resolution was set to 70,000, spray voltage was 3.5 kV, capillary temperature was 320°C, sheath gas flow was 40 AU, auxiliary gas flow was 10 AU, and collision energy was 20–40 eV (tandem MS, where applicable). Peptide identity was confirmed by matching the observed [M+H]+ peak to the theoretical molecular weight (Supplementary Methods **Lines 197–202**).

Comment 15: Line 382, What is the intensity of UV used in fabricating the PLA-HA scaffolds?

Response: The scaffold was fabricated using UV light at 365 nm, with an intensity of 10 mW/cm^2 for 60 seconds to initiate photocrosslinking. This information has now been added to the "Scaffold Preparation" section of the Materials and Methods **(Lines 419-420).**

Comment 16: The approval of the animal protocol by the Institutional Animal Care and Use Committee should be provided.

Response: We have now added the formal ethical approval statement to the Methods section. All animal experiments were conducted in accordance with the guidelines of Application for Laboratory Animal Welfare and Ethical review, School of Stomatology, Air Force Military Medical University, with the corresponding approval number: 2025 kq-006 **(Lines 458-460).**

Comment 17: Fig. 7 and 8, 4 weeks and 8 weeks should be labeled as the euthanization time points, not the intervals between events.

Response: We have revised Figures 7 and 8 and their corresponding legends to explicitly state that animals were euthanized and analyzed at 4 weeks and 8 weeks post-implantation. This change clarifies the experimental timeline and avoids ambiguity.

Comment 18: A critical discussion of the limitations of the study is recommended.

Response: In the revised manuscript **(Lines 381–389)**, we have expanded the final paragraph of the Results and Discussion section to explicitly address several limitations:

First, although our data support the concept that the scaffold enhances neurovascularized bone regeneration and is associated with increased ITGB1 expression, the precise molecular mechanisms underlying bidirectional ITGB1 activation remain incompletely defined. The current study demonstrates functional outcomes and correlative molecular changes, but does not establish direct causal signaling hierarchies at the intracellular level. Additional mechanistic studies, including pathway inhibition and receptor-binding specificity assays, will be necessary to fully dissect the downstream signaling network.

Second, while we incorporated sensory and sympathetic markers and a capsaicin-induced denervation model to improve specificity, the complex interactions among neural, vascular, and osteogenic compartments *in vivo* are multifactorial. Other cell types and signaling pathways may also contribute to the regenerative outcome, and these were not exhaustively explored.

Third, although the scaffold demonstrated promising regenerative effects in a rodent model, translation to large-animal models and eventual clinical settings requires further validation. Differences in scale, biomechanics, and immune responses may influence performance.

Finally, long-term safety, degradation kinetics, and potential immunogenicity beyond the studied time frame require further evaluation.

Bidirectional integrin β1 activation synergizes neurovascular coupling and enhances bone regeneration (NCOMMS-25-80494)

Responses to reviewers' comments

Reviewer #2:

Comment 2: I thank the authors for their thorough and timely revision of the manuscript. They have carefully addressed all my previous concerns, with detailed responses provided in the "Response to Reviewers" letter. The manuscript is now suitable for publication in Nature Communications.

The only remaining requirement is that the authors supply unmodified, fully uncropped original raw files for all Western blot data presented in the article.

Upon the author making minor revisions to address the aforementioned improvement, the manuscript could be accepted for publication.

Response: We greatly appreciate your recognition of our thorough revision. We provided the unmodified, fully uncropped original raw files for all Western blot data presented in Source Data. The Source Data has been uploaded and submitted together with the revised version. Thank you again for your time and constructive suggestions.

Reviewer #3:

Comment: Most of my concerns have been addressed now. One minor note is the Fig. 2A, the use of arrows between two reactive groups is confusing. The box on the IKVAV should be on an amine group, not on the methyl group. It is difficult to control which amines or carboxylic acids (side or terminal) to react. I recommend changing the related phases in the legend.

Response: We greatly appreciate your valuable suggestions. The conjugation between REDVGGG and IKVAV occurs between the carboxyl group of glycine in GGG and the amine group of isoleucine in IKVAV, forming a stable CONH amide bond. We have revised Figure 2A to show the chemical structure of REDVGGGIKVAV, with the amide bond highlighted in red to make the chemical reaction clearer. (Lines 841-843)
